# Identification and expression analysis of the *CqSnRK2* gene family and a functional study of the *CqSnRK2.12* gene in quinoa (*Chenopodium quinoa* Willd.)

**DOI:** 10.1186/s12864-022-08626-1

**Published:** 2022-05-24

**Authors:** Zhu Xiao-lin, Wang Bao-qiang, Wei Xiao-hong

**Affiliations:** 1 College of Agronomy, Gansu Agricultural University, Lanzhou, 730070 China; 2 College of Life Science and Technology, Gansu Agricultural University, Lanzhou, 730070 China; 3 Gansu Provincial Key Laboratory of Aridland Crop Science, Gansu Agricultural University, Lanzhou, 730070 China

**Keywords:** Quinoa, *SnRK2*, Biotic and abiotic stress, Subcellular localization, Transgenic Arabidopsis, Self-activation detection, Physiological Index

## Abstract

**Background:**

Sucrose non-fermenting 1 (SNF1)-associated protein kinase 2 (SnRK2) proteins belong to a relatively small family of plant-specific serine/threonine (Ser/Thr) protein kinases. SnRK2s participate in the abscisic acid (ABA) signaling pathway and play important roles in many biotic and abiotic stresses. At present, no *SnRK2* gene has been reported in quinoa, and the recently published genome for this species provides an opportunity to identify and characterize the *SnRK2* gene family.

**Results:**

We identified 13 *SnRK2* genes in the *C. quinoa* genome by bioinformatics analysis. Based on their phylogenetic relationships, these genes were divided into three subfamilies, similar to the situation in other plant species. Gene duplication analysis showed that there were seven pairs of homologous genes in the *CqSnRK2* family, and that purifying selection played an important role in the evolution of *SnRK2* genes. Gene structure analysis showed that the first exon in the *SnRK2* family genes has the same length as the last exon, and that *CqSnRK2* genes in the same subfamily have similar gene structures. Sequence analysis showed that the N-terminal region contains three highly conserved motifs. In addition, many kinds of cis-elements were identified in the promoter region of *CqSnRK2*, including those for hormone responses, stress responses, and tissue-specific expression. Transcription data analysis and qRT-PCR results showed that *CqSnRK2* has different expression patterns in roots, stems, and leaves, and responded to biotic and abiotic stresses such as low temperature, salt, drought, and abscisic acid (ABA). In addition, we found that the protein encoded by *CqSnRK2.12* was localized to the cytoplasm and nucleus, and there was no self-activation. The results of *CqSnRK2.12* overexpression showed that transgenic *Arabidopsis thaliana* lines had increased drought tolerance compared to the controls.

**Conclusion:**

The results of our study provide references for further studies on the evolution, function, and expression of the *SnRK2* gene family in quinoa.

**Supplementary Information:**

The online version contains supplementary material available at 10.1186/s12864-022-08626-1.

## Background

Plants are exposed to various abiotic stresses such as high salinity, drought, and low temperatures over the course of their growth and development [1]. These adverse conditions will seriously affect the yield and quality of crops, but plants have evolved adaptive mechanisms to protect themselves against a variety of environmental stresses that allow them respond to adverse conditions by regulating the production of beneficial substances in their cells or by initiating the expression of stress-related genes [2, 3]. Among these mechanisms are protein kinases and phosphatases that are the main components of intracellular signal transduction pathways which play key roles in the plant response to stresses [4]. Sucrose non-fermenting-1 (SNF1)-related protein kinases (SnRKs) are a family of serine/threonine (Ser/Thr) protein kinases widely found in plants. SnRKs are highly conserved and participate in various physiological processes [5, 6]. Based on the conserved kinase activity domains, SnRKs can be divided into three subfamilies; SnRK1, SnRK2, and SnRK3. SnRK1s are key components of cell signal transduction pathways, and the N-terminal region has a conserved catalytic domain, which regulates the energy balance of the cell by interacting with different forms of sugar in the cell. In addition, SnRK1s also respond to biological and abiotic stresses to regulates the growth and development of plants [6, 7]. SnRK1 proteins are highly homologous in structure and function to the AMPK protein in animals and the SNF1 protein in yeast. SnRK2 and SnRK3 are plant-specific protein kinases, which play key roles in plant resistance signaling [5].

The SnRK2 (Sucrose non-fermenting 1-related protein kinases 2) proteins are a relatively small plant-specific protein kinase family with typical N-terminal and C-terminal functional domains [8]. The N-terminal catalytic domain is highly conserved. The kinase domains present in AMPK and SNF1 are homologous and share 42%-46% similarity; the C-terminus is the regulatory region which is mainly composed of two subdomains called Domain I and Domain II [8, 9]. Domain I is composed of 30 amino acids and is present in all SnRK2 family members. It is mainly activated by adverse conditions, but does not depend on ABA. Domain II is composed of 40 amino acids, and is located close to the C-terminus, is unique to ABA-dependent SnRK2s, and is an essential structure in the response to ABA [8–11]. Compared with SnRK1, the C-terminus of SnRK2 lacks 140–160 amino acids and is relatively short. The distinctive feature of the C-terminus is an acidic patch structure rich in glutamic acid or aspartic acid (D/E) residues. Based on this, the SnRK2 family can be divided into the SnRK2a (Subclass I and Subclass II) and SnRK2b (Subclass III) subfamilies. The C-terminus of SnRK2a is rich in aspartic acid, while SnRK2b is rich in glutamic acid [9, 11, 12]. Studies have found that the C-terminal domain of SnRK2 is related to enzyme activation, ABA signal transmission, and protein interaction [13]. In addition, SnRK2 genes participate in ABA-dependent and ABA-independent signaling pathways to regulate osmotic stress, stomata opening and closing, seed germination, seedling development, and other plant growth processes [5, 14, 15]. Therefore, based on whether it is induced by ABA, the SnRK2 family can be divided into Groups I, II, III. Group I is not induced by ABA, Group II is slightly induced by ABA, and Group III is strongly induced by ABA. *AtSnRK2.1*, *AtSnRK2.4*, *AtSnRK2.5*, *AtSnRK2.9*, and *AtSnRK2.10* belong to Group I; *AtSnRK2.7* and *AtSnRK2.8* belong to Group II; and *AtSnRK2.2*, *AtSnRK2.3*, and *AtSnRK2.6* belong to Group III [16].

At present, there are 10–11 *SnRK2* gene family members in the monocotyledonous species *Brachypodium*, rice, wheat, corn, and sugarcane [10, 17–19], while in *Arabidopsis*, soybean, cotton, cherry, and other dicotyledonous species, the number of *SnRK2* gene family members ranges from 6 to 22 [9, 20–27]. The number of *SnRK2* gene family members in the bryophyte *Physcomitrella patens* is four [28]. These genes play a key role in the response of plants to abiotic stress. *SnRK2* mainly regulates downstream gene expression and protein activity through phosphorylation modification. The most representative of these is Ser175 of *AtSnRK2.6*. Its phosphorylation state is the key to kinase activity. In addition, all 10 *AtSnRK2* genes in Arabidopsis can be activated by osmotic stress, but only *AtSnRK2.9* can be activated by low temperature stress [29]. The expression of five *GhSnRK2* genes (*GhSnRK2.3/2.7/2.8/2.9/2.10*) in cotton increased significantly after salt stress and osmotic stress. All 10 *SnRK2* members in rice can be induced by hyperosmotic stress. Among them, transcription of *SAPK8*, *SAPK9*, and *SAPK10* is induced by ABA [30]. The transcriptional regulator *SAPK4* can regulate ion balance and plant growth and development, which is the process involved in plant salt stress [31]. There are 11 *SnRK2* family members in maize; among them, expression of *ZmSnRK2.2*, *ZmSnRK2.4*, *ZmSnRK2.5*, *ZmSnRK2.7*, and *ZmSnRK2.10* can be induced and activated by ABA. *ZmSnRK2.3* and *ZmSnRK2.6* can be strongly induced by NaCl. Under NaCl stress, transcription of *ZmSnRK2.3*, *ZmSnRK2.7* and *ZmSnRK2.11* was strongly induced, and transcription of *ZmSnRK2.5*, *ZmSnRK2.6*, and *ZmSnRK2.9* was inhibited by heat treatment [30]. Transcription of the wheat genes *TaSnRK2.3*, *TaSnRK2.4*, and *TaSnRK2.7* responds to drought, NaCl, low temperature, and cold stress. Overexpression of a *SnRK2* gene (*TaSRK2C1*) can significantly enhance the stress resistance of plants [32]. The *SoSnRK2.8*, *SoSnRK2.9* and *SoSnRK2.10* genes in sugarcane are strongly induced by ABA, and *SoSnRK2.1*, *SoSnRK2.2*, and *SoSnRK2.4* are strongly induced by low temperature, salt stress, and osmotic stress [17].

Quinoa (*Chenopodium quinoa* Willd.) is an annual dicotyledonous plant in the family Amaranthaceae that originated in the Andes Mountains of South America, quinoa seeds are rich in high-quality protein that contains a balance of essential amino acids that are required in the the human diet [33]. The grain is also gluten-free and is rich in vitamins, polyphenols, flavonoids, saponins and phytosterols, making it a complete nutritious food that can meet the basic nutritional needs of the human body [34]. The cultivation of quinoa outside of the Andean region has been heavily promoted by the United Nations Food and Agriculture Organization. Quinoa not only has extremely high nutritional value, but is also genetically diverse species with strong drought and salt tolerance that can be grown in marginal soils with pH values from 4.5 to 9.5 [35]. Exploring the biological basis of its excellent nutritional and agronomic characteristics has become a major focus of current crop research. In 2017, a high-quality reference genome of quinoa was released, which not only sparked a boom in quinoa research, but also promoted the breeding and cultivation of quinoa [34]. However, studies of the *SnRK2* gene family in quinoa have not been reported at present. Therefore, in this study, we identified 13 *SnRK2* family members from quinoa, and analyzed their basic physical and chemical properties, gene structure, and promoter cis-elements. In addition, the study of the *SnRK2* gene expression patterns in response to different stress treatments has given us a deeper understanding of this gene family gene in quinoa. The results of our study will provide a basis for future research on the functions of this gene family.

## Results

### Identification and basic properties analysis of quinoa *SnRK2* Gene

Based on protein homology searches, we identified 13 *SnRKs* genes from the quinoa genome, all of which encode proteins with complete serine/threonine protein kinase catalytic domains. These genes were named *CqSnRK2.1*-*CqSnRK2.13* (Table 1). The lengths of the proteins predicted to be encoded by the *CqSnRK2* gene family range from 133 aa (*CqSnRK2.5*) to 401aa (*CqSnRK2.1/CqSnRK2.13*). The predicted molecular weights are between 15,129.46 Da (*CqSnRK2.5*) and 45,652.03 Da (*CqSnRK2.5*). Ten of the proteins encoded by the *CqSnRK2* genes have a theoretical isoelectric point below 7, and are acidic proteins. The instability index is between 26.15 (*CqSnRK2.10*) and 48.06 (*CqSnRK2.1*). The average fatty acid index ranges from 79.42 (*CqSnRK2.10*) to 94.74 (*CqSnRK2.10*). The hydrophobicity index of all CqSnRK2 proteins is < 0, indicating that they are all hydrophilic protein. In addition, the subcellular localization prediction showed that nine of the proteins localize to the cytoplasm, and two of them (*CqSnRK2.6* and *CqSnRK2.9*) are predicted to be localized to the nucleus.

**Table 1:** Basic physicochemical properties of the proteins encoded by the 13 *SnRK2* family genes identified in the quinoa genome

Gene accession No	Gene	Size (aa)	Molecular weight (Da)	Isoelectric point	Instability index	Aliphatic index	GRAVY	Subcellular Localization
AUR62001966	*CqSnRK2.1*	401	45,652.03	4.79	48.06	89.98	-0.161	cytoplasm
AUR62003254	*CqSnRK2.2*	297	33,509.86	4.83	35.71	84.68	-0.335	cytoplasm
AUR62003840	*CqSnRK2.3*	323	36,822.89	4.73	47.63	90.56	-0.188	cytoplasm
AUR62004814	*CqSnRK2.4*	345	38,858.75	9.29	41.33	79.42	-0.367	microbody
AUR62004815	*CqSnRK2.5*	133	15,129.46	9.07	44.58	87.22	-0.410	cytoplasm
AUR62007175	*CqSnRK2.6*	341	38,559.81	5.38	32.26	84.05	-0.445	nucleus
AUR62011423	*CqSnRK2.7*	347	39,411.57	4.87	41.70	89.88	-0.370	cytoplasm
AUR62016637	*CqSnRK2.8*	347	39,397.59	4.91	43.10	90.17	-0.361	cytoplasm
AUR62018830	*CqSnRK2.9*	341	38,505.82	5.38	33.52	84.34	-0.412	nucleus
AUR62018955	*CqSnRK2.10*	215	23,893.67	6.44	26.15	94.74	-0.099	cytoplasm
AUR62027216	*CqSnRK2.11*	316	35,593.22	4.97	37.74	84.21	-0.317	cytoplasm
AUR62027801	*CqSnRK2.12*	293	32,804.47	5.05	37.55	92.12	-0.237	cytoplasm
AUR62036033	*CqSnRK2.13*	401	45,611.71	8.90	39.43	85.31	-0.195	cytoplasm

### Phylogenetic analysis of the *SnRK2* family in quinoa

**Fig. 1:**
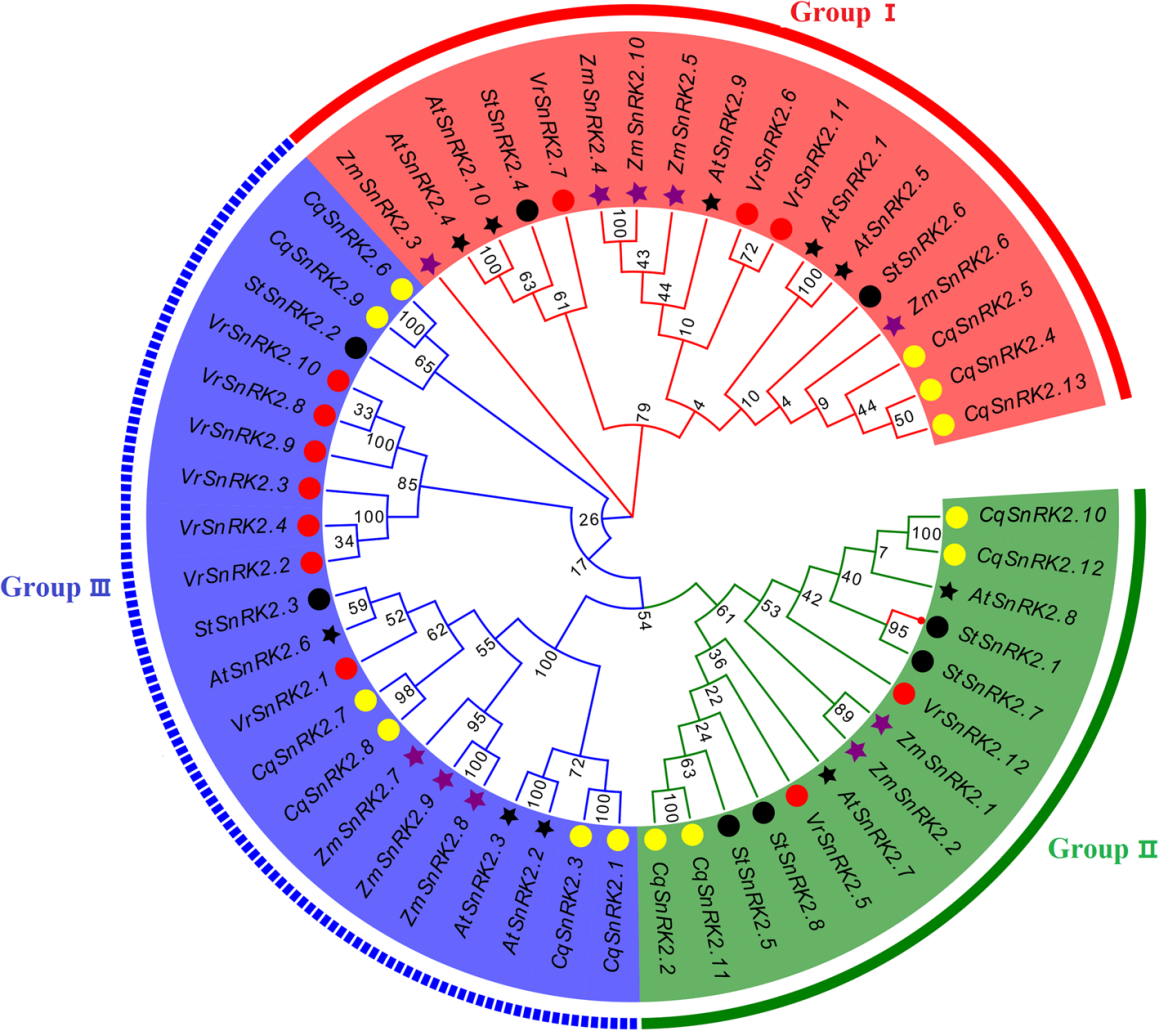
Phylogenetic analysis of *SnRK2* protein families in *Arabidopsis thaliana*, potato, maize, mung bean and quinoa. The phylogenetic tree was constructed using the Maximum Likelihood method by MEGA X. The bootstrap values of 1000 replicates were calculated at each node. The model was p-distance and the pattern among Lineages was Same (Homogeneous). The gaps and missing data treatment were complete deletion. Different colored arcs indicate different subfamilies. Black solid circles, red solid circles, yellow solid circles, black solid stars, and purple solid stars represent SnRK2 proteins from potatoes, mung bean, quinoa, Arabidopsis, and corn, respectively

In order to understand the evolutionary relationships of the proteins encoded by the *CqSnRK2* genes, we used 53 SnRK2 protein sequences from five different plant species to construct a phylogenetic tree (Fig. 1, Table S1). Of these, 13 CqSnRK2 proteins were from quinoa, 10 ZmSnRK2 proteins were from maize, 10 AtSnRK2 proteins were from Arabidopsis, 8 StSnRK2 proteins were from potato, and 12 VrSnRK2 proteins were from mung bean. As shown in Fig. 1, the phylogenetic analysis grouped the predicted SnRK2 proteins into three major clades (Groups I, II, and III). We found that there are 13 pairs of paralogous genes (five pairs of *CqSnRK2*, three pairs of *CqSnRK2*, two pairs of *AtSnRK2*, two pairs of *ZmSnRK2*, and 1 pair of *StSnRK2*), four pairs of orthologous genes (*ZmSnRK2.1*/*AtSnRK2.9*, *StSnRK2.2*/*AtSnRK2.8*, *ZmSnRK2.2*/*AtSnRK2.7*, and *StSnRK2.4*/*VrSnRK2.6*). A previous study showed that expression of *AtSnRK2.2*, *AtSnRK2.3*, and *AtSnRK2.6* is activated by ABA and that these genes participate in ABA signal transduction [9], indicating that six quinoa genes (*CqSnRK2.1*, *CqSnRK2.3*, *CqSnRK2.7*, and *CqSnRK2.8* in Group III, and *CqSnRK2.10* and *CqSnRK2.12* in Group II) may also participate in the ABA response.

### Chromosomal locations and duplication of *SnRK2* genes in quinoa

In order to further study the genetic divergence and gene duplication events in the *CqSnRK2* gene family, we determined the chromosomal locations of the 13 *CqSnRK2* genes. The results (Fig.S1) showed that except for two *CqSnRK2* genes located on Chr05 (B), the remaining 11 *CqSnRK2* genes were evenly distributed on all 11 chromosomes.

In addition, gene duplication is considered to be an important force that drives evolution. Studies have shown that duplicate genes are produced at a very high rate, about 0.01 times per gene per million years. There are generally two types of replication, tandem repetition and fragment repetition. Tandem duplications occur on the same chromosome, while segmental duplications can occur on the same or different chromosomes. In this study, we found seven pairs of duplicated genes (Table 2) that can be classified as segmental duplication events. In addition, the non-synonymous (K_A_) and synonymous (K_S_) substitution rates between the seven repeated gene pairs was calculated. Ka/Ks = 1 indicates "neutral or no selection", Ka/Ks < 1 indicates "negative or purifying selection"; and Ka/Ks > 1 indicates "positive selection". In this study, the Ka/Ks ratios of the seven pairs of repeated genes were all < 1, indicating that these gene pairs have undergone purifying selection.

**Table 2:** The Ka/Ks ratios and predicted duplication dates for duplicated *SnRK2* genes in quinoa

Duplicated SnRK2 gene1	Duplicated SnRK2 gene2	Ka	Ks	Ka/Ks	Date(mya)T = Ks/2λ	Selective pressure	Duplicate type
*CqSnRK2.1*	*CqSnRK2.3*	0.017	0.130	0.130	4.341	Purifying selection	Segmental
*CqSnRK2.4*	*CqSnRK2.6*	0.275	3.528	0.078	117.596	Purifying selection	Segmental
*CqSnRK2.4*	*CqSnRK2.3*	0.333	3.505	0.095	116.821	Purifying selection	Segmental
*CqSnRK2.8*	*CqSnRK2.7*	0.005	0.092	0.054	3.078	Purifying selection	Segmental
*CqSnRK2.9*	*CqSnRK2.6*	0.006	0.110	0.052	3.654	Purifying selection	Segmental
*CqSnRK2.10*	*CqSnRK2.12*	0.054	0.210	0.256	6.992	Purifying selection	Segmental
*CqSnRK2.11*	*CqSnRK2.2*	0.009	0.120	0.074	3.989	Purifying selection	Segmental

### Exon/intron structures of the *CqSnRK2* genes and conserved motifs in the predicted CqSnRK2 proteins

**Fig. 2:**
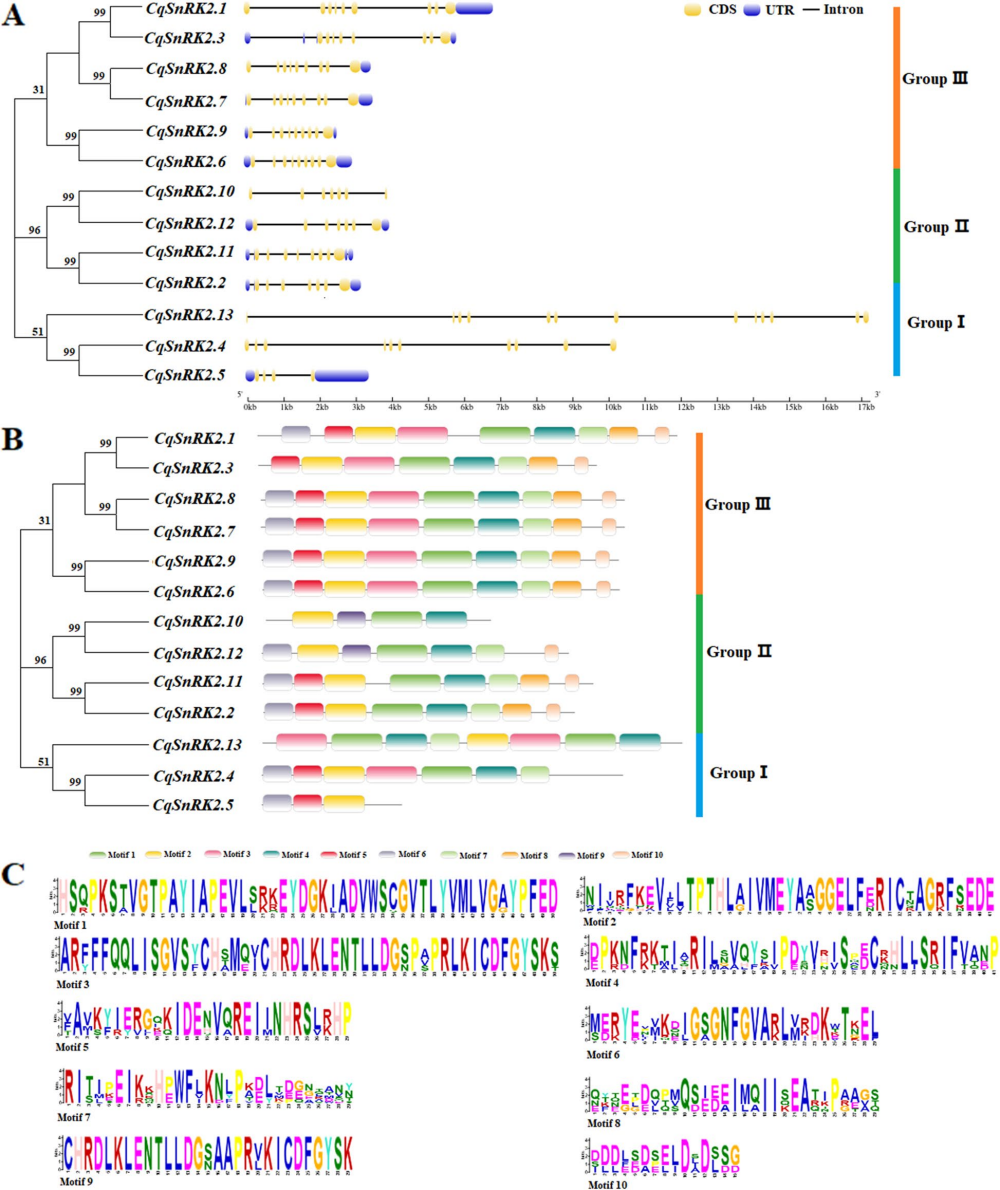
Gene structure and conserved motif of *SnRK2* gene in quinoa. **A** Intron–exon gene structure; Blue boxes represent untranslated 5'- and 3'- regions, yellow boxes represent exons, and black lines represent introns. **B** conservative basic motifs; Different colors represent different motifs. **C** Basic composition of 10 conserved motifs

The intron–exon structure can provide a more systematic and comprehensive understanding of the conserved features and the evolution of the gene family. In this study, we found that most *CqSnRK2* genes have a highly conserved distribution of exons and introns. In eight of the *CqSnRK2* genes (except for *CqSnRK2.1*, *CqSnRK2.4*, *CqSnRK2.5*, *CqSnRK2.10*, and *CqSnRK2.13*) the first exon has the same length as the last exon, and the *CqSnRK2* genes in the same subfamilies have similar gene structures (Fig. 2A, Table S2). The 13 *CqSnRK2* genes all have between 3 and 12 introns and 4–13 exons. Ten of the *CqSnRK2* genes have 7–9 exons. In addition, eight *CqSnRK2* genes have untranslated regions (UTRs) at the 3' and 5' ends, three genes (*CqSnRK2.4*, *CqSnRK2.10*, and *CqSnRK2.13*) have no terminal UTRs, and two genes (*CqSnRK2.1* and *CqSnRK2.8*) have no UTR at the 5’ end.

The SnRK2 proteins have highly conserved N-terminal regions and variable C-terminal regions. Therefore, we analyzed the conserved motifs present in the CqSnRK2 proteins and found a total of 10 conserved motifs (Fig. 2B,C). Motifs 2, 5, and 6 are found at the N-terminus and are highly conserved in most CqSnRK2 proteins (Fig. 2C); motif 2 is present in all CqSnRK2 proteins, motifs 1 and 4 are present in 12 CqSnRK2 proteins (except CqSnRK2.5), motif 3 is present in eight CqSnRK2 proteins (except CqSnRK2.2, CqSnRK2.5, CqSnRK2.10, CqSnRK2.11, and CqSnRK2.12), motif 8 is present in eight CqSnRK2 proteins (except CqSnRK2.4, CqSnRK2.5, CqSnRK2.10, CqSnRK2.12, and CqSnRK2.13), and motif 9 is only present in the two CqSnRK2 proteins CqSnRK2.10 and CqSnRK2.12. In addition, CqSnRK2 proteins in the same subfamilies also have similar motif structures.

### Construction of a protein interaction network and cis-acting element analysis

**Fig. 3:**
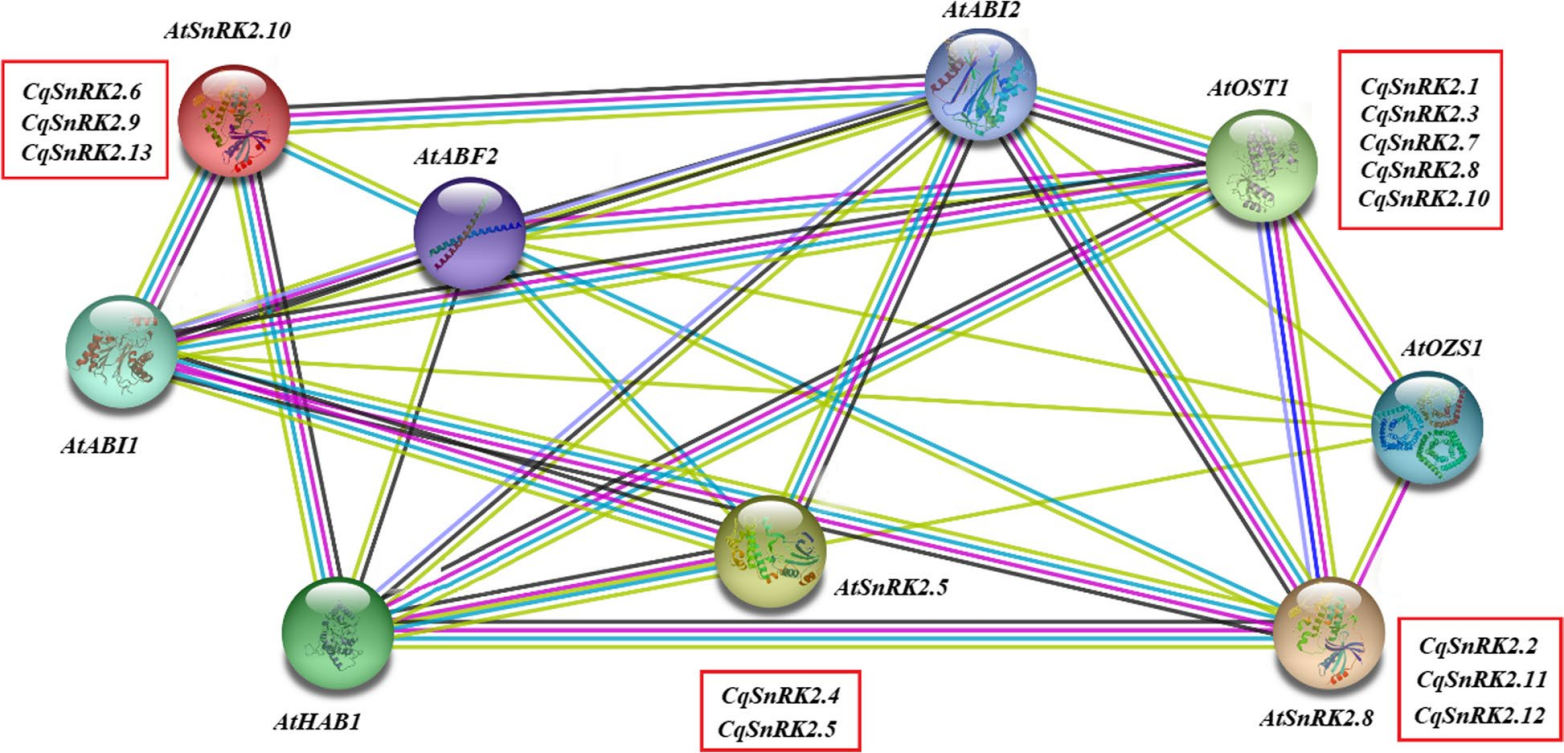
The prediction of the interaction network of CqSnRK2 proteins based on the interactions of their orthologs in Arabidopsis

The study of protein interaction networks not only helps to understand the functions of the individual proteins that comprise the network, but also helps to explain the biological functions of most proteins in the cell, and identifies key proteins in the protein interaction network to enable the study of proteins related to stress resistance. Therefore, in order to further understand the interaction of *SnRK2* genes in quinoa, in this study, we constructed a protein interaction network diagram based on the homologous proteins from Arabidopsis (Fig. 3). Among them, we found that *AtSnRK2.5* (*CqSnRK2.4* and *CqSnRK2.5*) and *AtSnRK2.10* (*CqSnRK2.6*, *CqSnRK2.9* and *CqSnRK2.13*) participate in ABA-independent abiotic stress resistance and regulate the osmotic stress response [36]. *AtSnRK2.8* (*CqSnRK2.2*, *CqSnRK2.11*, and *CqSnRK2.12*) is involved in hormone signal transduction (salicylic acid) [37]. *AtOST1* (Open Stomatal1) regulates ABA-mediated stomatal closure and is a protein kinase that functions upstream of the ROS (reactive oxygen species) formation pathway. OST1 domain 2 interacts with PP2C-type phosphatases (ABI1 and ABI2). In Arabidopsis, this interaction between PP2C and OST1 integrates ABA and osmotic stress signals and regulates the closure of stomata [38]. Therefore, *CqSnRK2.1*, *CqSnRK2.3*, *CqSnRK2.7*, *CqSnRK2.8*, and *CqSnRK2.10* may also have similar functions in this study. In addition, it has been shown that SnRK2 can directly phosphorylate ABF proteins and the ABI1 protein [39]. In this study, SnRK2 protein can interact with proteins such as ABF, ABI, and HABI to perform the corresponding functions.

**Fig. 4:**
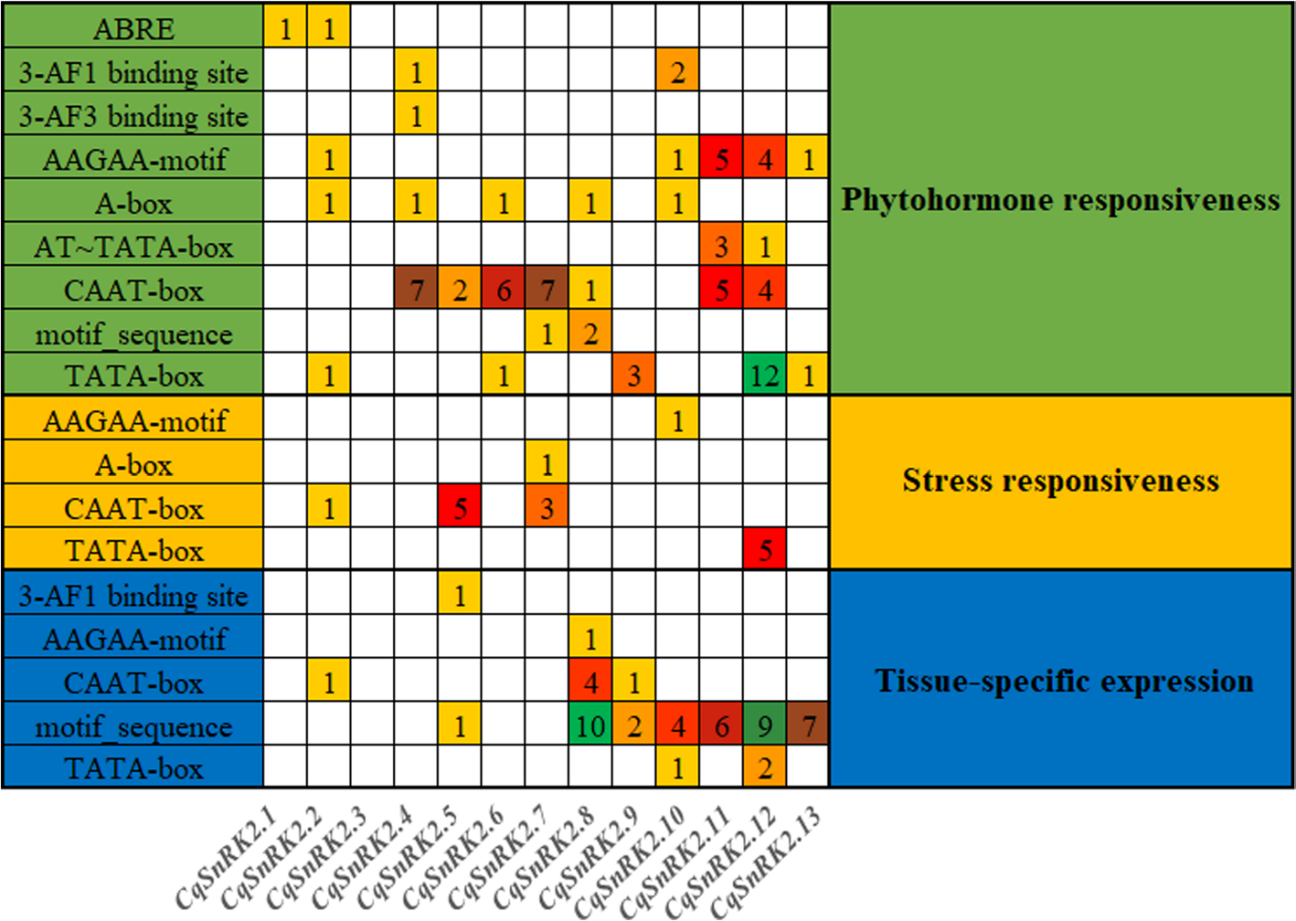
The cis-regulatory element in the promoter region of *SnRK2* genes of quinoa. The colors and numbers on the grid indicate the number of different cis-regulatory element in the *SnRK2* genes

Cis-acting elements located in the promoter region of a gene have multiple functions and play key roles in regulating the function of the gene. Therefore, we analyzed the promoter sequence upstream of the *CqSnRK2* genes. Eighteen cis-acting element elements with different sequences were identified upstream of the 13 *CqSnRK2* genes (Fig. 4, Table S3). Further analysis revealed that these elements are mainly involved in hormone responses, the stress response, and tissue-specific expression. In terms of hormone responses, eight cis-elements respond to ABA (ABRE, 3-AF1 binding site, AAGAA-motif, A-box, AT-TATA-box, CAAT-box, motif-sequence, and TATA-box). One cis element responds to methyl jasmonate (CAAT-box), one cis element responds to auxin (CAAT-box), and two cis elements respond to gibberellin and salicylic acid (CAAT-box and TATA-box). The promoters of 12 of the *CqSnRK2* genes (all except *CqSnRK2.3*) contain ABA response elements. Three cis-elements (AAGAA-motif, A-box, and CAAT-box) are involved in inducing the drought response, the CAAT-box element responds to low temperature, and the CAAT-box and TATA-box elements are involved in stress and defense responses. In terms of tissue-specific expression, the 3-AF1 binding site, TATA-box, AAGAA-motif, and CAAT-box elements are related to endosperm expression, motif-sequence is related to anaerobic induction and zein metabolism, and the TATA-box starts in the root effect. In addition, we found that the promoter regions of *CqSnRK2.8* (19), *CqSnRK2.11* (19), and *CqSnRK2.12* (37) contained the most cis-elements. The majority of the *CqSnRK2* (9) genes contained 6–12 cis-elements,. The *CqSnRK2.1* and *CqSnRK2.3* promoters contained the fewest cis-elements—1 and 0, respectively.

### Expression patterns of *CqSnRK2* genes in different tissues and stress treatments

Using transcriptome data, we evaluated the expression patterns of the *CqSnRK2* genes in the different stress treatments (drought, low temperature, heat, and salt) and in the different tissues and organs (Fig.S2, Table S4). We found that, compared with the control, almost all the genes examined in the four different treatments had different levels of expression in the roots and shoot tips. Compared with the control, five genes (*CqSnRK2.1*, *CqSnRK2.3*, *CqSnRK2.5*, *CqSnRK2.12*, and *CqSnRK2.13*) showed significantly up-regulated expression after drought treatment, and in another five genes (*CqSnRK2.2*, *CqSnRK2.7*, *CqSnRK2.8*, *CqSnRK2.9*, and *CqSnRK2.11*), the expression levels showed significant down-regulation after drought treatment. Compared with the control, we found that the expression of one or a few genes differed significantly in individual treatments. For example, *CqSnRK2.2*, *CqSnRK2.6*, and *CqSnRK2.9* were significantly down-regulated under salt stress and *CqSnRK2.9* was significantly down-regulated in response to heat stress. The expression patterns in different tissues and organs indicated that some genes (*CqSnRK2.1*, *CqSnRK2.3*, *CqSnRK2.5*, *CqSnRK2.7*, *CqSnRK2.10*, *CqSnRK2.12*, and *CqSnRK2.13*) are highly expressed in all of the tissues and organs examined in this study. In addition, 10 of the *CqSnRF2* genes (except *CqSnRK2.1*, *CqSnRK2.3*, and *CqSnRK2.10*) showed low expression levels in the fruits of yellow bitter quinoa. The expression of 10 *CqSnRK2* genes (except *CqSnRK2.8*, *CqSnRK2.10*, and *CqSnRK2.12*) in stems was significantly higher than in leaves, indicating that the expression of some *CqSnRK2* genes showed tissue specificity.

### Expression profiling of quinoa *SnRK2* genes under drought, low temperature, NaCl, PEG, and ABA treatments

**Fig. 5:**
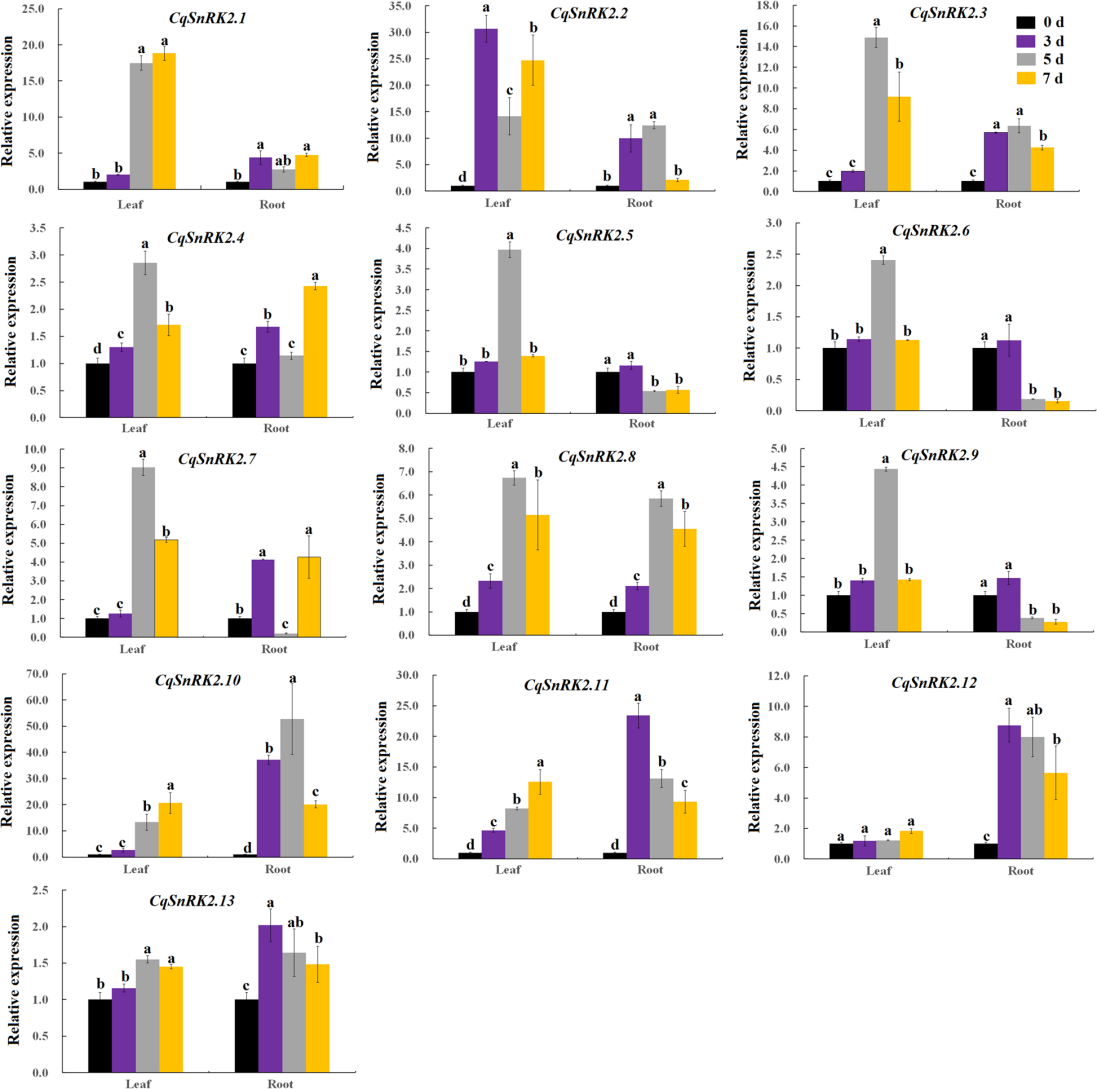
qRT-PCR analysis of 13 *CqSnRK2* gene expression patterns in leaves and roots of quinoa under drought stress. *TUB-9* of quinoa as an internal reference gene, the relative expression level was calculated by **2**^**−ΔΔCt**^, and different lowercase letters represented significance at 0.05 level (*P* < 0.05). The data are the mean ± SE of three independent biological samples, the vertical line represents the standard deviation, and the primer information is in Table S4

We analyzed the expression of all *CqSnRK2* family genes in the roots and leaves of quinoa under drought stress using qRT-PCR (Fig. 5, Table S5-Table S6). In leaves, most genes (except *CqSnRK2.12*) were up-regulated after drought treatment, and we observed that the relative expression of most genes (except *CqSnRK2.2*, *CqSnRK2.8*, and *CqSnRK2.11*) was not significantly different compared to the control three days after drought stress. The expression of *CqSnRK2.2* (increasing first, then decreasing, and finally increasing) reached its maximum at 3 d after drought stress, and the expression of other *CqSnRK2* genes peaked at 5 d (*CqSnRK2.3*, *CqSnRK2.4*, *CqSnRK2.5*, *CqSnRK2.6*, *CqSnRK2.7*, *CqSnRK2.8*, and *CqSnRK2.9*) or 7 d (*CqSnRK2.1*, *CqSnRK2.10*, *CqSnRK2.11* and *CqSnRK2.13*) after drought stress, indicating that as the degree of drought deepens, the expression of *CqSnRK2* family genes in leaves is activated to respond to the drought stress. In roots, the expression of *CqSnRK2* family genes is not as strong as in leaves. The expression of some genes (*CqSnRK2.5*, *CqSnRK2.6*, *CqSnRK2.9*, *CqSnRK2.11*, *CqSnRK2.12*, and *CqSNRK2.13*) reached the maximum at 3 d or 6 d (*CqSnRK2.2*, *CqSnRK2.3*, *CqSnRK2.8*, and *CqSNRK2.10*) under drought stress. The relative expression of three *CqSnRK2* genes (*CqSnRK2.5*, *CqSnRK2.6*, and *CqSnRK2.9*) at 5 and 7 days after drought stress were significantly lower than in the control. In addition, the relative expression of *CqSnRK2.10* gene was significantly higher than in the control (50-fold) at 5 days after drought stress.

**Fig. 6:**
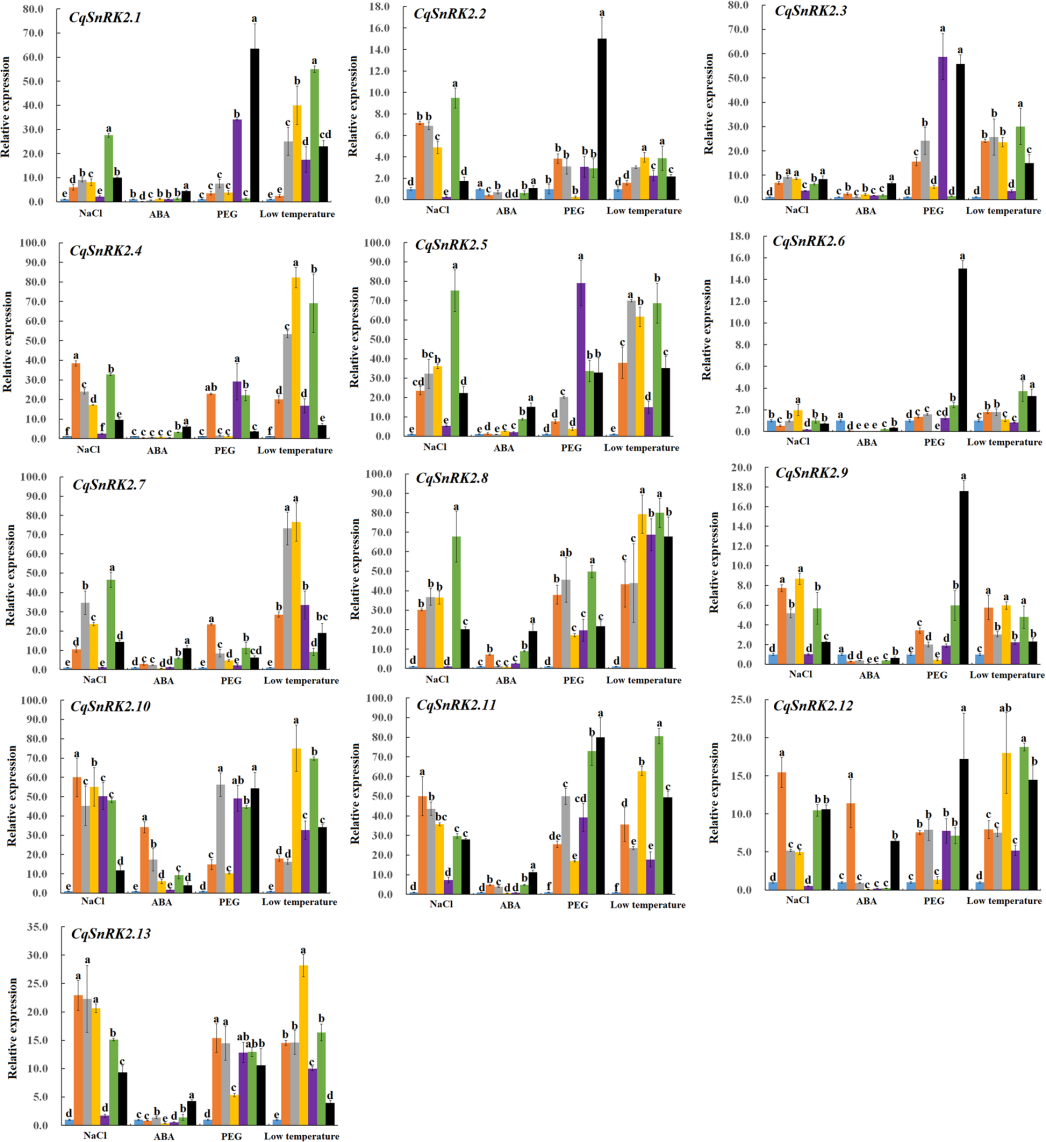
qRT-PCR analysis of 13 *CqSnRK2* gene expression patterns under different stress (200 mmol/L NaCl, 200umol/L ABA, 20% PEG and 4℃ low temperature). *TUB-9* of quinoa as an internal reference gene, the relative expression level was calculated by 2^−ΔΔCt^, and different lowercase letters represented significance at 0.05 level (*P* < 0.05). The data are the mean ± SE of three independent biological samples, the vertical line represents the standard deviation

Expression of quinoa *SnRK2* family genes is induced by PEG6000, low temperature, ABA, and NaCl treatments (Fig. 6, Table S7). Analysis of *SnRK2* gene expression at different time points over a 48 h period revealed that the relative expression levels of *SnRK2* genes were significantly different at different treatment times. The results of qRT-PCR analysis showed that after the 200 mmol/L NaCl treatment, the relative expression of five genes (*CqSnRK2.4*, *CqSnRK2.10*, *CqSnRK2.11*, *CqSnRK2.12*, and *CqSnRK2.13*) reached the maximum at 3 h after treatment, and the expression of *CqSnRK2.10* was up-regulated to 60-fold that of the 0 h control. Five genes (*CqSNRK2.1*, *CqSnRK2.2*, *CqSnRK2.5*, *CqSnRK2.7*, and *CqSnRK2.8*) showed the highest relative expression levels at 24 h after treatment. The expression level of *CqSnRK2.5* was 75 times higher than at 0 h. In the 200 μmol/L ABA treatment, we found that the relative expression of most *CqSnRK2* genes changed little after ABA treatment, and the relative expression of seven genes (*CqSnRK2.1*-*CqSnRK2.4*, *CqSnRK2.6*, *CqSnRK2.9*, and *CqSnRK2.13*) was tenfold that of the control at all time points after treatment. The relative expression of nine genes (*CqSnRK2.1*-*CqSnRK2.5*, *CqSnRK2.7*, *CqSnRK2.8*, *CqSnRK2.11*, and *CqSnRK2.13*) peaked at 48 h after treatment, and there were significant differences compared with expression at 0 h. *CqSnRK2.10* and *CqSnRK2.12* are a duplicated gene pair, and the relative expression level of both reached the maximum at 3 h after treatment. *CqSnRK2.10* had the largest relative up-regulation, which was 34.14-fold higher than expression at 0 h; *CqSnRK2.6* expression peaked at 9 h after treatment, and was 1.98-fold higher that at 0 h. *CqSnRK2.9* was down-regulated at all time points after ABA treatment, which implies that this gene plays a negative regulatory role in plant abiotic stress. After 20% PEG treatment, there were significant differences in the expression of the *CqSnRK2* family genes and the control. We found that the expression of seven genes (*CqSnRK2.1*, *CqSnRK2.2*, *CqSnRK2.6*, *CqSnRK2.9*, *CqSnRK2.10*, *CqSnRK2.11*, and *CqSnRK2.12*) reached their maximum at 48 h after treatment. Among these genes, the relative expression of *CqSnRK2.11* was the most highly up-regulated, and was 80-fold higher than expression at 0 h. In addition, we found that the expression of specific genes was significantly different from the control at certain time points after PEG treatment (*CqSnRK2.1* gene was significantly different from the control at 12 h and 48 h after the treatment, and *CqSnRK2.2* expression was only significantly different from the control at 48 h after treatment), and expression of some genes, such as *CqSnRK2.8*, *CqSnRK2.10*, *CqSnRK2.11*, *CqSnRK2.12*, and *CqSnRK2.13* was significantly different from the control at multiple time points after PEG treatment. In the 4℃ low temperature treatment, the relative expression levels of 12 genes reached the maximum at 9 h (*CqSnRK2.2*, *CqSnRK2.4*, *CqSnRK2.7*, *CqSnRK2.8*, *CqSnRK2.9*, *CqSnRK2.10*, and *CqSnRK2.13*) or 24 h (*CqSnRK2.1*, *CqSnRK2.3, CqSnRK2.6, CqSnRK2.11*, and *CqSnRK2.12*) after treatment, and the expression of *CqSnRK2.5* at 9 h and 24 h was also relatively high compared to the control. Thus, our results show that the relative expression almost all of the *CqSnRK2* genes showed an increasing expression pattern from 0 h-9 h, and the relative expression of genes between 9 and 48 h showed a decrease-increase–decrease expression pattern. Expression levels of 10 *CqSnRK2* genes (*CqSnRK2.1*, *CqSnRK2.4*, *CqSnRK2.5*, *CqSnRK2.7-CqSnRK2.13*) were significantly different from the controls at all time points after low temperature treatment. The relative expression level of *CqSnRK2.8* at each time point after low temperature treatment was very high (at least 40-fold that of the control), leading us to hypothesize that this gene plays a key role in the response of plants to low temperature stress.

### Subcellular localization of *CqSnRK2.12* and detection of self-activation in yeast

**Fig. 7:**
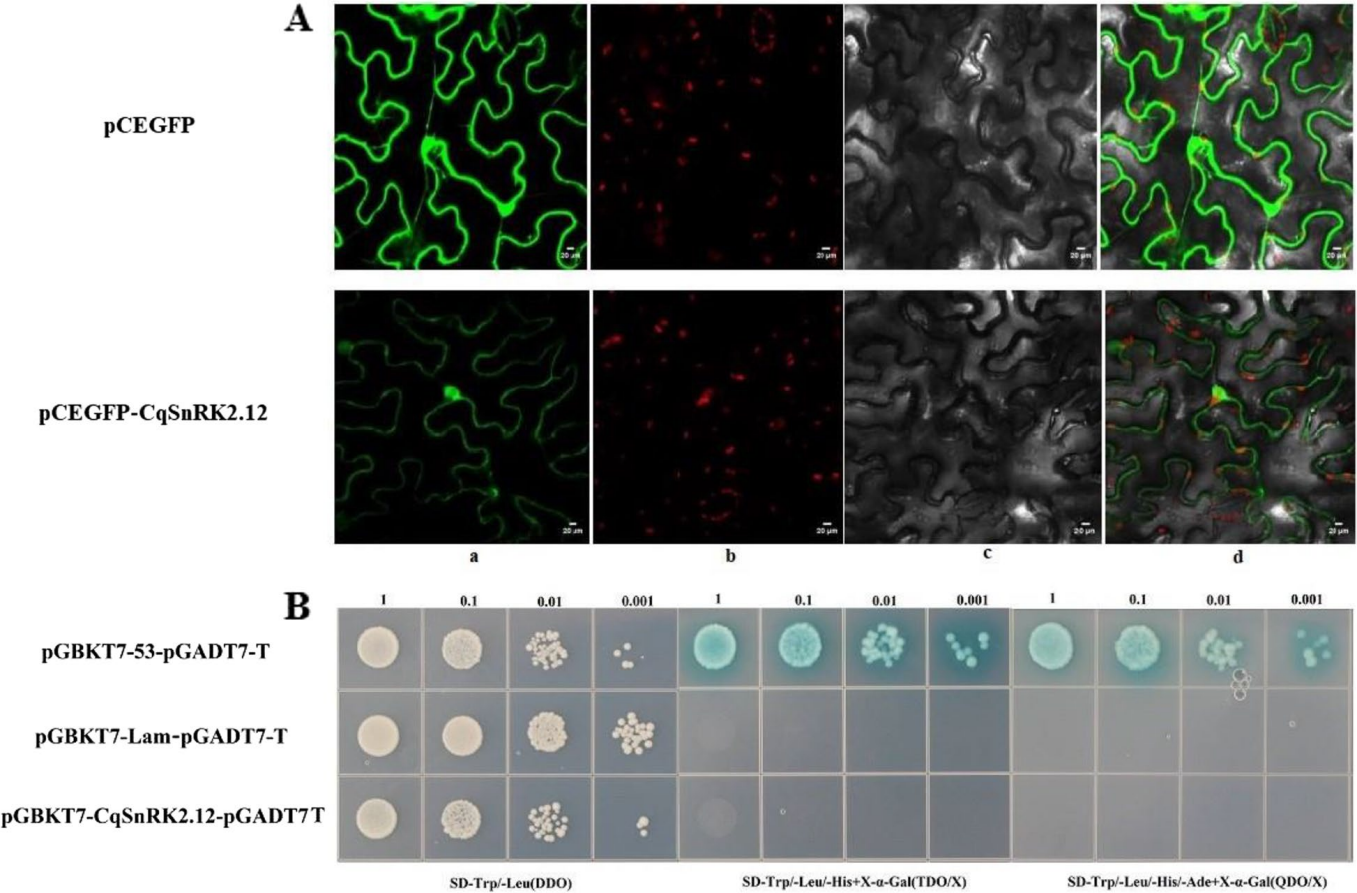
Subcellular localization and yeast self-activation detection. **A**: Localization of *CqSnRK2.12* in tobacco leaves, CqSNRK2.12-GFP represents the gene and GFP represents the control that is an empty vector. a: pCEGFP fluorescence signal in the dark field; b: Tobacco leaves transformed with 35 S: pC1302 -EGFP and 35 S: GFP-CqSNRK2.12 in green pictures for the finding of green fluorescent protein (GFP); c: cell morphology under bright field; d: Merged figures for the finding of light and dark images. The images were taken by LEICA DMi8, Japan fluorescence microscopy. **B**: Auto activation state of Yeast Two Hybrid Interaction of *CqSnRK2.12* gene. pGBKT7-53-pGADT7-T: Positive Control; pGBKT7-Lam-pGADT7-T: Negative control; pGBKT7-CqSnRK2.12-pGADT7-T: Experimental group

The DNA construct carrying the chimeric *CqSnRK2.12***-***GFP* gene in Agrobacterium was infiltrated into tobacco leaves and the fluorescent signal was observed in the epidermal cells by laser scanning confocal microscopy. As shown in Fig. 7A, the CqSnRK2.12-GFP fusion protein was expressed in the cytoplasm and the nucleus, which was consistent with the predicted localization for the CqSnRK2.12 protein. In addition, transcriptional self-activation of the protein was detected. The results of the bait vector self-activation test (Fig. 7B) showed that yeast cells containing the positive control vector could grow on all three media. The negative control grew only on SD/-Trp/-Leu medium, and yeast cells containing PGBKT7-CqSnRK2.12 + pGADT7 were able to grow on SD/-Trp/-Leu medium. However, there was no colony growth on SD/-Leu/-Trp/-His/X-α-Gal and SD-Leu/-Trp/-His/-Ade/X-α-Gal media. These results showed that the CqSnRK2.12 protein did not have transcriptional self-activation.

### Overexpression of *CqSnRK2.12* increased salt and drought stress tolerance in Arabidopsis

**Fig. 8:**
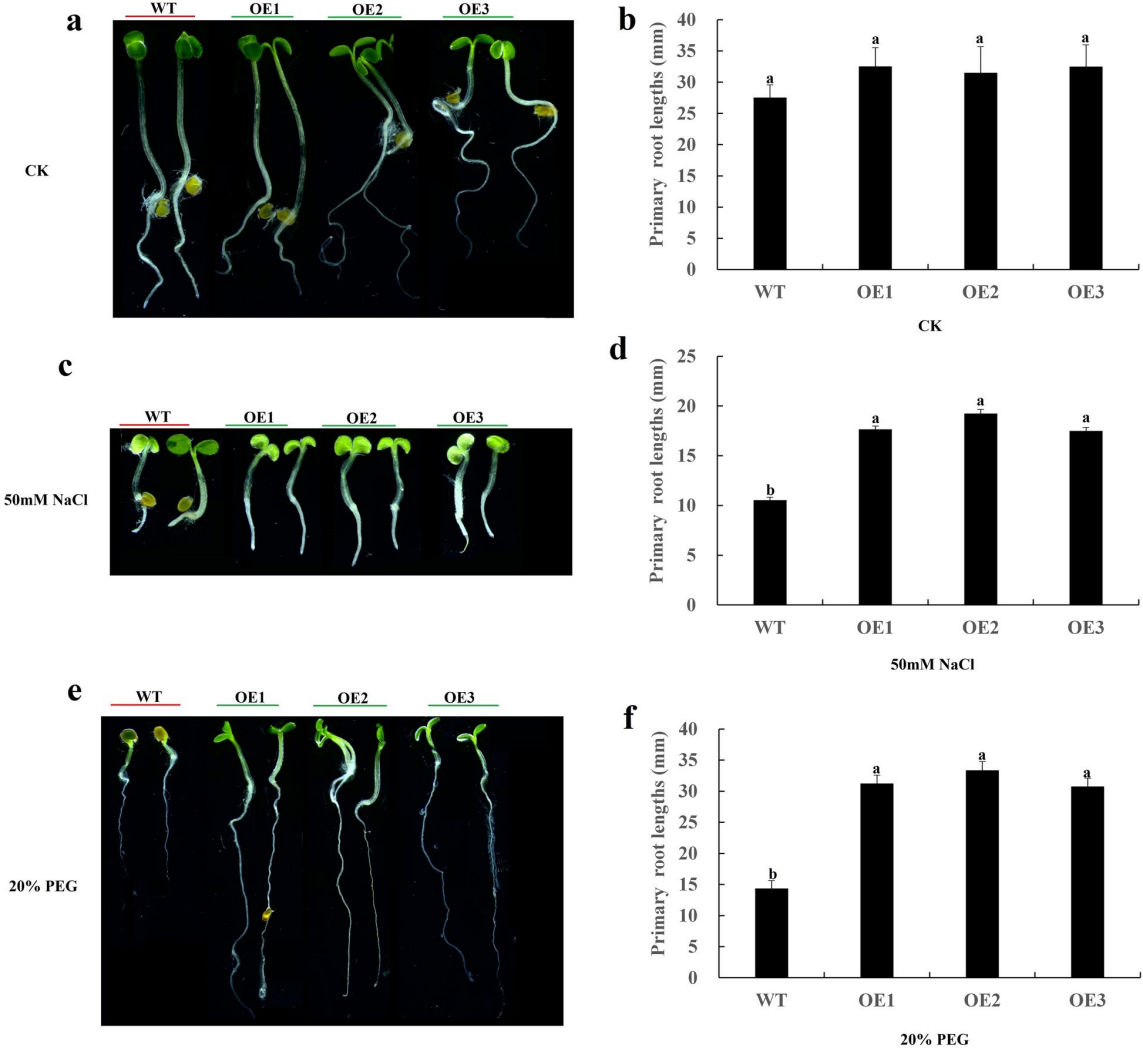
Effects of salt and drought stress on root length of wild-type (WT) and Arabidopsis overexpressing *CqSnRK2.12* (OE-1, OE-2, and OE-3). **a** Sterile water for 7 days. **b** Root length statistics in Sterile water. **c** 50 mM NaCl for seven days. **d** Root length statistics in 50 mM NaCl for seven days. **e** 20% PEG for 7 days. **f** Root length statistics in 20% PEG for 7 days. Different lowercase letters represented significance at 0.05 level (*P* < 0.05). The data are the mean ± SE of three independent biological samples, the vertical line represents the standard deviation

In order to further study the function of *CqSNRK2.12* gene, we introduced *CqSnRK2.12* into *Arabidopsis thaliana* by *Agrobacterium tumefaciens*-mediated transformation, and obtained homozygous T_3_-generation over-expression lines (OE-1, OE-2, OE-3). Under normal growth conditions, we found that there was no significant difference in growth between the transgenic OE lines and the WT (Fig. 8a, b). However, in the 50 mM NaCl and 20% PEG treatments, we observed significant differences in growth between the transgenic and WT plants. In the 50 mM NaCl treatment (Fig. 8c, d), the root lengths of transgenic and WT plants were inhibited compared with the CK, (no salt) but the root lengths of plants overexpressing *CqSnRK2.12* were significantly longer than in WT plants; the average root length in the transgenic plants was 1.718-fold that of wild plants. In the 20% PEG treatment (Fig. 8e, f), the average root length of the OE transgenic plants was 2.21-fold that of WT plants. These results indicate that *CqSnRK2.12* participates in the regulation of drought and salt stress responses, and that over-expression of *CqSNRK2.12* could induce drought and salt tolerance in transgenic Arabidopsis plants.

## Discussion

When plants encounter harmful external environments, they can adapt to stresses through various morphological, physiological, and molecular reactions. In these processes, the phosphorylation of protein kinases induced by stress plays an important role in plant sensing and the response to environmental stress [40]. The *SnRK2* gene family encodes plant-specific Ser/Thr protein kinases that play important roles in a variety of plant signal transduction and stress responses (drought resistance, cold resistance, and salt-alkali resistance) [22]. In this study, we identified 13 *SnRK2* genes the quinoa (*Chenopodium quinoa*) genome. The number of *SnRK2* gene family members in the monocots *Brachypodium* (10), rice (10), wheat (10), maize (11), and sugarcane (10) are between 10 and 11, indicating that this gene family is highly conserved in monocotyledonous plants. The number of *SnRK2* family genes is more variable in dicotyledons, ranging between 6 and 22; examples are Arabidopsis (10), soybean (22), cotton (20), cherry (6), and *Populus pilosa* (12) [9, 19–28]. The higher numbers in some species indicate that this gene family is prone to gene duplication in dicots. The number of *SnRK2* gene family members in the moss species *Physcomitrium patens* is four. Compared with bryophytes, the number of SnRK2 protein in angiosperms has increased significantly, which may be related to polyploidization and gene duplication in angiosperms, and may also be because the genes in the *SnRK2* family expanded due to the whole genome duplication events that occurred after these plant lineages diverged. At the same time, we can clearly see that the number of *SnRK2* genes shows an evolutionary trend from aquatic algae to terrestrial mosses and ferns to angiosperms, which is consistent with a previous report [5]. A phylogenetic analysis showed that the proteins encoded by the *SnRK2* genes group in three clades in this study. Previous studies showed that the SnRK2 proteins from multiple plants species also cluster into three discrete groups [20, 21, 27, 41]. Sequence alignments showed that the N-terminus of the SnRK2 proteins contains a relatively conserved protein kinase catalytic domain, while the C-terminus is highly specific and consists of an activation domain that responds to abiotic stress and an ABA acidic amino acid regulatory domain. Subcellular localization prediction of the quinoa SnRK2 proteins indicates that the members of this family are mainly localized to the cytoplasm and nucleus, while CqSnRK2.4 is found in the microbody, and may be involved in the glycolysis pathway. CqSnRK2.13 is localized to the plasma membrane, and its function could be related to the synthesis of membrane lipoproteins.

Gene structure prediction shows that most members of the quinoa *SnRK2* gene family have 7–9 exons, and the distribution of exons within each subfamily is similar. There are large differences in the sequences of *SnRK2* genes between the different subfamilies which is mainly due to the differences in the lengths of introns and other non-coding regions, which is consistent with findings in cotton and rice [10, 24]. Motif analysis showed that the order of the motifs is conserved in each subfamily, and that most of the motif positions within the same subfamily are also basically the same. This may indicate that genes in the same subfamily have similar functions. Only motif 9 is quite different, indicating that the motifs of the same subfamily have similar functions. Wheat SnRK2 proteins show a high degree of evolutionary conservation, which is consistent with research results in mung bean [23]. These results point out the high-level evolutionary conservation of the SnRK2 protein family in plants. During evolution, most gene families expand through continuous duplication and differentiation. On the one hand, gene duplication and differentiation provide abundant original genetic material, but on the other hand, these processes can also produce most of the genetic variation which makes it possible to adapt to changing environmental conditions through natural selection. Gene duplication analysis in this study identified seven pairs of duplicated genes in the *CqSnRK2* family, which led to an expansion of the number of gene family members. Duplication events including segmental duplication and tandem repeats have played critical roles in the expansion of gene families in plants [42, 43]. In addition, based on the chromosomal location and changes,such as mutations in gene structure, new members may show diverse functions [44, 45]. Our findings show that some duplicated *CqSnRK2* genes in quinoa experienced purifying selection and have evolved new functions.

When plants are subjected to abiotic stresses such as drought, high temperature, low temperature, and salinity, the signals related to the perception of these stresses will be converted into responses through a series of signal transduction pathways [1]. These responses will activate the transcription of genes that encode stress-related transcription factors that then bind to the cis-acting regulatory elements in the promoters of the corresponding genes, initiating the expression of these genes in the specific response to the stress [1, 2, 46]. Therefore, the functional study of cis-acting elements present in stress resistance-related genes is particularly important for revealing the mechanisms that control plant stress resistance. The promoter regions of *SnRK2* genes usually contain cis-acting elements related to hormones and stress responses, such as the ABA response element ABRE, the gibberellic acid response element GARE-motif, the methyl jasmonate response element CGTCA-motif, the heat shock response element HSE, the low temperature stress response element LTRE, the drought response element DRE, the stress response element TC-rich repeats, and the MYB transcription factor binding element MBS. These cis-actig elements are related to hormone signal transduction and stress responses [36], and the *SnRK2* gene family plays a crucial role in the plant response to abiotic stress and ABA-dependent plant development [47, 48]. In this study, we found that *CqSnRK2.11* (19 cis-acting elements) and *CqSnRK2.12* (37 cis-acting elements) contain the AAGAA-motif, AT-TATA-box, CAAT-box, and motif-sequence elements, and most *CqSnRK2* genes contain 6–12 cis-acting elements (between 3–6 element types). We can speculate that most of the genes in this gene family are involved in the stress response mechanisms to cold, drought, and salt tolerance. Among them, the *CqSnRK2.12* gene may be involved in a variety of stress responses. In addition, we found that 12 *CqSnRK2* genes (all except *CqSnRK2.3*) contain ABA response elements, which may be an important reason why the transcription of genes in this family is strongly induced by ABA.

Our study found that the relative expression levels of the *CqSnRK2.2*, *CqSnRK2.5* and *CqSnRK2.11* genes were high in stems, flowers, and mature seeds, and very low in leaves and developing seeds. Expression of *CqSnRK2.6* and *CqSnRK2.9* was almost undetectable in inflorescences and apical meristems, while *CqSnRK2.5* and *CqSnRK2.13* were highly expressed in both the inflorescence and apical meristem. The expression levels of most *CqSnRK2* genes were higher in white quinoa flowers than in yellow quinoa flowers, and the expression levels of all *CqSnRK2* genes in white quinoa fruits were higher than those in yellow quinoa fruits. It can be seen that the *SnRK2* gene family members are expressed in various tissues of quinoa, but that the expression levels in vary in the different tissues t, showing a certain degree of tissue/organ specificity. In addition, the experimental results also reflect the differences between varieties. Kobayashi [10] used Northern blot hybridization to detect the expression of the *SnRK2* gene family *SAPK* in rice tissues such as leaves, leaf sheaths, and roots under different stress conditions, and showed that the expression of members of this gene family was different in different tissues. The conclusions of this study are consistent with the results of our study. The specificity of the expression of *SnRK2* gene family members in different tissues under normal conditions may be because they mediate different physiological metabolic processes in plants under normal growth conditions. Studies have shown that SnRK2 proteins regulate seed dormancy and germination, sex differentiation, root system morphogenesis, flowering, fruit ripening, yield formation, plant height, and other processes that affect the growth and development of plants [49]. This may be the reason for the differential expression of *SnRK2* genes in different tissues found in this study.

The SnRK2 proteins comprise a family of protein kinases that participate in the responses to abiotic stresses in plants. SnRK2 proteins play important roles in the normal growth and development of plants, responding to ABA signals as well as abiotic stress caused by drought, high salt, low temperature, and salinity [5]. Previous studies have shown that overexpression of the wheat *TaSnRK2.4* gene can significantly improve plant tolerance to salt, drought, and freezing damage [50]. Overexpression of *AtSnRK2.8* significantly improved drought resistance [51]. In this study, the expression of quinoa genes homologous to *AtSNRK2.8* (*CqSnRK2.2*, *CqSnRK2.11*, and *CqSnRK2.12*) increased significantly in response to drought and PEG treatments. These three quinoa *CqSnRK2* genes belong to the Group II subfamily, and their expression is slightly induced by ABA. Therefore, their function in the drought response may be mediated through the ABA signaling pathway. At the same time, the promoter regions of these three genes contain multiple ABA response elements, especially *CqSnRK2.12*, which contains 21 ABA response elements. However, it is interesting that the expression of *CqSnRK2.12* under drought stress was not as high as that of *CqSnRK2.2* and *CqSnRK2.11*, suggesting that there may be other uncharacterized cis-elements or unknown mechanisms associated with stress that are involved in the regulation of these genes. *AtSnRK2.6*, also known as *OST1*, belongs to the Group III subfamily and is strongly induced by ABA. This gene is the main positive regulator of ABA signal transduction and stomatal control [49]. Silencing of *AtSnRK2.6* expression leads to damage to stomatal closure and serious water loss in Arabidopsis leaves, indicating that *AtSnRK2.6* mainly plays a positive regulatory role in drought stress. In this study, six quinoa genes (*CqSnRK2.1*, *CqSnRK2.3*, and *CqSnRK2.6*-*CqSnRK2.9*) belonging to Group III were strongly expressed in response to drought stress. The high expression levels of these genes may induce the ABA signaling pathway and result in stomatal closure, thus enhancing drought resistance in quinoa. In addition, in Arabidopsis, *AtSnRK2.2*, *AtSnRK2.3*, and *AtSnRK2.6* are usually activated by ABA and the proteins encoded by these gene can phosphorylate ABRE binding factors. Therefore, they are important for the activation of ABA-responsive genes. The *AAPK* gene in broad bean is also a member of the *SnRK2* family, and it is induced by ABA in guard cells to regulate stomatal closure [52]. The overexpression of *SPK3* in soybean is induced by exogenous ABA, which increases the plant response to hyperosmotic stress [53]. *SnRK2.10* in *Populus tomentosa* interacts with ABI1, AHG1, and AHG3 to participate in ABA signal transduction, and responds to drought and salt stress [27]. The protein interaction network predicted in this study shows that quinoa SnRK2 family proteins can also interact with ABI1, ABI2, and ABF2 and interact with HAB1 to respond to abiotic stresses such as drought, salinity, and low temperature. Studies have shown that in Arabidopsis, except for *AtSnRK2.9*, other *SnRK2* protein kinase genes can be activated by substances that regulate osmotic pressure, such as sucrose, mannitol, sorbitol, or NaCl [5]. This result was also confirmed in our study, and the expression of the gene family members changed significantly in response to the three abiotic stresses. Studies have shown that transgenic plants overexpressing the maize *SAPK8* and rice *SAPK4* genes have significantly enhanced salt tolerance compared to WT controls [31]. Previous studies have shown that *SnRK2* Group III genes are important components of ABA signaling pathways and can be induced by exogenous ABA [10]. In this study, we found that transcription of most of the Group I, Group II, and Group III genes can be induced by ABA, a result that is inconsistent with previous studies in Arabidopsis and rice [10], which indicates that quinoa has a complex ABA-dependent signal transduction pathway. Studies have shown that all *AtSnRK2* family members in Arabidopsis are not induced by low temperature, but some *SnRK2* genes have a strong response to cold, including *PKABA1*, *TaSnRK2.3*, *TaSnRK2.4*, *ZmSnRK2.3*, *ZmSnRK2.7*, and *GsAPK* [25]. In contrast, our results also show that expression of all *CqSnRK2s* in quinoa can be induced by low temperature and are significantly up-regulated, but it is not entirely clear whether their expression contributes to conferring frost resistance and enhanced plant survival; therefore, this finding provides a new perspective on the involvement of *SnRK2* genes in the response to low temperature stress. At the same time, we found an interesting phenomenon that the expression patterns of partially duplicated genes were slightly different or significantly different, such as the duplicated gene pair CqSnRK2.3/CqSnRK2.4, which presented different expression patterns after PEG stress. It indicated that these two genes Functional divergence occurred during evolution, and this functional divergence may be related to homeopathic elements contained in the promoter region, which was also reported in Wang's study [46]. Although the *CqSnRK2* family genes are involved in the responses to a variety of abiotic stresses, the effects of these stresses on expression do not fully reflect their function, because their activation is more directly and rapidly regulated after translation. Therefore, more studies on the functional characterization of *CqSnRK2* genes are needed.

## Conclusion

In this study, we systematically identified the SnRK2 family from the quinoa genome. We used bioinformatics method to describe the physical and chemical properties, gene structure, protein interactions and promoter elements of 13 *SnRK2* genes. Gene structure and motif analyses corroborated the phylogenetic analysis results. The cis-elements found in the promoters of the *CqSnRK2* gene were found to be associated with phytohormones and abiotic stresses. RT-qPCR analysis revealed that *CqSnRK2.4-CqSnRK2.5*, *CqSnRK2.8*, *CqSnRK2.10- CqSnRK2.13* were significantly up-regulated in response to low temperature, salt and drought stress. In addition, the overexpression of *CqSnRK2.12* increased salt and drought stress tolerance in Arabidopsis.

## Methods

### Materials and treatments

The test material used was the quinoa cultivar ‘Longliu No. 2 that was obtained as seed from the Gansu Academy of Agricultural Sciences. Seed was sown in the greenhouse of the College of Life Science and Technology of Gansu Agricultural University in March 2021. Quinoa seeds of the same size and fullness that were free of pests and diseases were selected and soaked in warm water for 20 min, after which they were sown in pots containing 2 kg of sandy loam soil. Thirty seeds were sown per pot and were covered with vermiculite, after which the pots were transferred to a greenhouse and grown at a temperature of 20–25℃ with front and back ventilation, natural light, and normal watering. When the seedlings had grown to the 6–8 leaf stage (about one months after sowing), they were thinned, leaving 10 seedlings in each pot. After the seedlings are colonized, they were subjected to five stress treatments: (1) PEG stress: 20% PEG; (2) Salt stress: 200 mmol/L NaCl; (3) Low temperature stress: 4℃; (4) Hormone treatment: 200 μmol/L ABA. The treatments were performed at 0 h when the light period started, and quinoa leaves were sampled at 0, 3, 6, 9, 12, 24, and 48 h after the initiation of the treatments. (5) Drought treatment: plants were not watered, and quinoa leaves and roots were sampled at 0, 3, 5, and 7 days after watering ceased. The collected materials were placed at -80 °C prior to their use in the subsequent experiments. There were three replicates for each treatment.

### RNA extraction and cDNA synthesis

Total RNA was extracted using AG RNA e x Pro Reagent (Beijing, China). The extracted RNA was the template for cDNA synthesis using the Evo M-MLV RT Kit (Beijing, China) to reverse transcribe 0.5–2 μg of purified total RNA into first-strand cDNA for gene amplification and real-time quantitative PCR (qRT-PCR) assays.

### Identification and cloning of the Quinoa SnRK2 protein kinase family

In this study, the deduced amino acid sequence of the Arabidopsis SnRK2 protein was used as the query sequence. Genes encoding homologous proteins were identified in the quinoa genome [34], and the quinoa *SnRK2* gene family members were initially screened as candidate genes. We then used HMMER (https://www.ebi.ac.uk/Tools/hmmer/) to search for conserved sequences and to eliminate candidate genes for proteins that do not contain the specific SnRK2 kinase domain (registration number: PF00069.25). The conserved CqSnRK2 domains were determined using the tools NCBI-CDD (https://www.ncbi.nlm.nih.gov/cdd/), SMART(http://smart.embl.de/) and Pfam (http://pfam.xfam.org/). Finally, 13 *CqSnRK2* genes were identified.

### Construction of a phylogenetic tree of the Quinoa SnRK2 protein family and related information analysis

After screening, the quinoa gene family that encodes SnRK2 proteins was analyzed using the Expasy (https://web.expasy.org/) online tool ProtParam to compute the physical and chemical properties such as the number of amino acids, theoretical isoelectric point, molecular weight, and hydrophobicity index. The PSORT Prediction (http://psort1.hgc.jp/form.html) tool was used to predict the subcellular locations of the SnRK2 proteins [54]. A phylogenetic tree was then constructed with the Maximum Likelihood method as implemented in MEGA X using the default values [55]. The confidence levels of the individual branches were estimated by bootstrapping using 1,000 replicates. The quinoa GFF3 annotation file and genome sequence were imported into the software TBtools [56] to draw the exon–intron structural diagrams. The conserved protein motifs were analyzed with MEME (http://meme-suite.org/tools/meme) [57]. TBtools was used to locate genes on chromosomes from the GFF annotation files [56]. We detected duplicate gene pairs using the plant genome replication database server http://chibba.agtec.uga.edu/duplication/index/locket). Clustal W software was used to predict the amino acid sequences of the partially repeated *CqSnRK2* genes [55]. TBtools software calculates the Ka and Ks values of duplicated genes [56]. The protein interaction network was constructed based on orthologous proteins from the model plant *Arabidopsis thaliana*, and the network diagram was drawn by String (https://cn.string-db.org/) [58]. The 2,000 bp of DNA sequence upstream of the *CqSnRK2* genes was extracted by TBtools [56] as the promoter regions, and PlantCARE (http://bioinfor-matics.psb.ugent.be/webtools/plantcare/html/) [59] was used to analyze the cis-elements in these promoter regions.

### Expression pattern analysis and quantitative real-time fluorescence PCR analysis

The expression data for the different tissues and organs of quinoa and the different stress treatments (drought, salt, and low temperature) were downloaded from the NCBI BioProject database (accession numbers PRJNA394651 and PRJNA306026, respectively).

Fragments per kilobase of exon model per million mapped reads (FPKM) values were used to represent the expression levels of the *SnRK2* genes. We selected the transcriptome data of genes belonging to the SnRK2 transcription factor family. Using TBtools software (https://github.com/CJChen/TBtools) [56], log_10_ FPKM values were calculated, a heat map was drawn, and the *SnRK2* genes in quinoa tissues and the different treatments were then analyzed [60]. Quantitative real-time fluorescence PCR (qRT-PCR) was performed on an Mx3005P Real-Time PCR System (Stratagene, USA) and the SYBR Green Premix Pro Taq HS qPCR Kit (Beijing, China) were used to determine gene expression levels. The primers for qRT-PCR were designed using the Takara online tool (https://www.takarabio.com/). The internal reference gene control was *TUB-9* (Table S4). The amplification system consisted of 1 μL cDNA, 0.5 μl upstream and downstream primers (10umol/L), 10 μl reaction mix, and 8 μl ddH_2_O in a total volume of 20 μL. The reaction conditions consisted of a single denaturation step at 95 °C for 30 s, followed by 40 cycles of 95 °C for 5 s and 60 °C for 30 s. The fluorescence curve and the melting curve were analyzed after the reactions were completed. The relative expression of each gene was calculated using the 2^−∆∆CT^ method [61].

### Subcellular localization of the CqSnRK2.12 protein and transcriptional activation of *CqSnRK2.12*

Using homologous recombination, the cloned *CqSnRK2.12* gene was transferred to an expression vector containing the gene for green fluorescent protein (GFP) to construct GV1300::CqSnRK2.12-GFP, and the vector plasmid was transferred to Agrobacterium GV1300 by electroporation. Positive clones were selected and cultured in liquid medium. The plasmid-containing Agrobacterium strains were infiltrated into tobacco (*Nicotiana benthamiana*) leaves. Expression of the CqSnRK2-12: GFP fusion protein in leaf epidermal cells was observed and imaged using a confocal laser scanning microscope (LSCM).

The *CqSnRK2.12* gene was cloned into the plasmid pGBKT7 at the *Nco* I site in frame with the yeast GAL4 bonding domain (BD). The bacterial colonies were screened by PCR using 3’ BD and T7 primers, and the amplified PCR products were sequenced. A plasmid extraction kit was used to extract the plasmid DNA from an *E. coli* culture containing the correct recombinant plasmid. The pGBKT7-*CqSNRK2.12* vector was transferred into yeast strain AH109 and cultured on an SD/TRP plate at 30 °C. Colonies that grew on the plate after 2–3 d were screened by PCR. The recombinant strain was grown in liquid medium, and 10 μl aliquots of a dilution series (10^–1^, 10^–2^, 10^–3^, 10^–4^, and 10^–5^) were spotted onto different media and the growth of the yeast was observed at 30 °C.

### Generation of transgenic *Arabidopsis thaliana* plants and determination of related physiological parameters

The *CqSnRK2.12* gene (without the terminator sequence) was transferred into the pCAMBIA-1302-EGFP vector by homologous recombination, and the recombinant plasmid was transformed into *E. coli* DH5α. After screening on solid medium containing kanamycin resistance, positive clones were selected and sequenced. The recombinant Plasmid pCAMBIA-1302: CqSnRK2.12 was transferred into *Agrobacterium tumefaciens* GV3101. Positive clones were identified, and plants of the wild-type (WT) *A. thaliana* ecotype Columbia (Col-0; seeds obtained from the Gansu Academy of Agricultural Sciences) were transformed by the floral dip method. The T_0_-generation transgenic seeds were sterilized in 6.25% NaClO solution for 15 min, washed with sterile ddH_2_O three times, and sown on MS (Murashige-Skoog) medium containing 50 μg/mL hygromycin B for selection of T_1_-generation transformants. Genomic DNA was extracted from the T_1_-generation plants to determine whether the *CqSnRK2.12* gene construct was inserted into the Arabidopsis genome. Total RNA was also extracted from leaves and used in qRT-PCR assays.

PCR detection: Genomic DNA was extracted from leaves of kanamycin resistant transgenic plants and WT *A. thaliana* Col-0 plants; the CqSnRK2.12-OE recombinant plasmid DNA was used as the positive control and Col-0 DNA was the negative control. Reference primer pairs HYG-417BP (F: 5’-AAATCCGCGTGCACGAGGT-3’; R: 5’-TCCTTGTTGTTCCTTCGGTTCTGTGTGTGCA-3’) and HYG-501BP (F: 5’-GAGATACCGCCAGTC-3’; R: 5’-CAAGACCCTGCGAACCGA-3’) were used to identify transgenic plants by PCR amplification. T_3_-generation transgenic plants were identified by hygromycin (150 mg/L) selection for subsequent experimental analysis. For the salt stress and drought treatments, seeds of the transgenic and WT lines were sterilized and cultured on 20% PEG and 50 mM NaCl, respectively. The plant phenotypes were observed and the root length was measured after 7 days of growth on MS medium at 22^◦^C.

## Statistical analysis

The statistical analysis was performed using Microsoft Excel 2010 and SPSS 25.0. The means among various groups were compared by Duncan’s multiple range tests. The data were analysed and are expressed as the means ± stand- ard deviations (SDs), and *P* < 0.05 indicated significance differences.

## Supplementary Information

**Additional file 1.** 

**Additional file 2.** 

**Additional file 3:**  **Table S1.**  The 53 SnRK2 gene-coding protein sequence information in this study.

**Additional file 4:** **Table S2.** Amino acid sequence information of 13 SnRK2 genes.

**Additional file 5:** **Table S3.** Cis-acting elements in the promoter region of CqSnRK2 genes.

**Additional file 6:** **Table S4.** The values of 13 CqSnRK2 genes in 9 tissues and 5 tretments downloaded from NCBI.

**Additional file 7:** **Table S5.** The primer designed for qRT-PCR.

**Additional file 8:** **Table S6.** Relative expression of 13 genes by qRT-PCR in drought stress.

**Additional file 9:** **Table S7.** Relative expression of 13 genes by qRT-PCR in different stress. 

## Data Availability

The reference genome assembly used for data analysis was obtained from National Center for Biotechnology Information (NCBI) BioProject PRJNA675125. The raw transcriptome data generated and analysed in this study deposited in SRA of the NCBI under accession number PRJNA394651 and PRJNA306026. The datasets analysed during this study are included in this published article and its supplementary information files.

## Abbreviations

ABA: Abscisic acid
qRT-PCR: Quantitative RT-PCR
SnRK2: Sucrose non-fermentative 1-associated protein kinase 2
UTR: Untranslated region
GFP: Green fluorescent protein

## Declarations

### Statement on guidelines

All experimental studies and experimental materials involved in this research are in full compliance with relevant institutional, national and international guidelines and legislation.

## References

[1] Hadiarto T, Tran L (2011). Progress studies of drought-responsive genes in rice. Plant Cell Rep.

[2] Bohnert HJ, Gong Q, Li P, Ma S (2006). Unraveling abiotic stress tolerance mechanisms–getting genomics going. Curr Opin Plant Biol.

[3] Miyakawa T, Fujita Y, Yamaguchi-Shinozaki K, Tanokura M (2013). Structure and function of abscisic acid receptors. Trends Plant Sci.

[4] Ludwig AA, Tina R, Jones J (2004). CDPK-mediated signalling pathways: specificity and cross-talk. J Exp Bot.

[5] Kulik A, Wawer I, Krzywińska E, Bucholc M, Dobrowolska GY (2011). SnRK2 Protein Kinases—Key Regulators of Plant Response to Abiotic Stresses. Omics.

[6] Fàbregas N, Yoshida T, Fernie AR (2020). Role of Raf-like kinases in SnRK2 activation and osmotic stress response in plants. Nat Commun.

[7] Halford NG, Hardie DG (1998). SNF1-related protein kinases: global regulators of carbon metabolism in plants?. Plant Mol Biol.

[8] Huai J, Wang M, He J, Zheng J, Dong Z, Lv H, Zhao J, Wang G (2008). Cloning and characterization of the SnRK2 gene family from *Zea mays*. Plant Cell Rep.

[9] Guo D, Li HL, Zhu JH, Wang Y, Peng SQ (2017). Genome-wide identification, characterization, and expression analysis of SnRK2 family in Hevea brasiliensis. Tree Genet Genomes.

[10] Kobayashi Y, Yamamoto S, Minami H, Hattori KT (2004). Differential Activation of the Rice Sucrose Nonfermenting1-Related Protein Kinase2 Family by Hyperosmotic Stress and Abscisic Acid. Plant Cell.

[11] Yoshida R, Umezawa T, Mizoguchi T, Takahashi S, Takahashi F, Shinozaki K (2006). The regulatory domain of SRK2E/OST1/ SnRK2.6 interacts with ABI1 and integrates abscisic acid (ABA) and osmotic stress signals controlling stomatal closure in Arabidopsis. J Biol Chem.

[12] Saha J, Chatterjee C, Sengupta A, Gupta K, Gupta B (2014). Genome-wide analysis and evolutionary study of sucrose non-fermenting 1-related protein kinase 2 (SnRK2) gene family members in Arabidopsis and Oryza. Comput Biol Chem.

[13] Vlad F, Rubio S, Rodrigues A, Sirichandra C, Belin C, Robert N, Leung J, Rodriguez PL, Laurière C, Merlot S (2009). Protein Phosphatases 2C Regulate the Activation of the Snf1-Related Kinase OST1 by Abscisic Acid in Arabidopsis. Plant Cell.

[14] Mao X, Zhang H, Tian S, Chang X, Jing R (2010). TaSnRK2.4, an SNF1-type serine/threonine protein kinase of wheat (Triticum aestivum L.), confers enhanced multistress tolerance in Arabidopsis. J Exp Bot.

[15] Coello P, Hey SJ, Halford NG (2011). The sucrose non-fermenting-1-related (SnRK) family of protein kinases: potential for manipulation to improve stress tolerance and increase yield. J Exp Bot.

[16] McLoughlin F, Galvan-Ampudia CS, Julkowska MM, Caarls L, van der Does D, Laurière C, Munnik T, Haring MA, Testerink C (2012). The Snf1-related protein kinases SnRK2.4 and SnRK2.10 are involved in maintenance of root system architecture during salt stress. Plant J.

[17] Li C, Nong Q, Xie J, Wang Z, Liang Q, Solanki MK, Malviya MK, Liu X, Li Y, Htun R, Wei J, Li Y (2017). Molecular Characterization and Co-expression Analysis of the SnRK2 Gene Family in Sugarcane (Saccharum officinarum L.). Scientific Rep..

[18] Wang L, Hu W, Sun J, Liang X, Yang X, Wei S, Wang X, Zhou Y, Xiao Q, Yang G, He G (2015). Genome-wide analysis of SnRK gene family in Brachypodium distachyon and functional characterization of BdSnRK2.9. Plant Science.

[19] Zhang H, Li W, Mao X, Jing R, Jia H (2016). Differential Activation of the Wheat *SnRK2* Family by Abiotic Stresses. Front Plant Sci.

[20] Bai J, Mao J, Yang H, Khan A, Fan A, Liu S (2021). Sucrose non-ferment 1 related protein kinase 2 (SnRK2) genes could mediate the stress responses in potato (Solanum tuberosum L.). BMC Genetics.

[21] Boneh U, Biton I, Schwartz A, Ben-Ari G (2012). Characterization of the ABA signal transduction pathway in *Vitis vinifera*. Plant Sci.

[22] Boudsocq M, Barbier-Brygoo H, Lauriere C (2004). Identification of nine sucrose nonfermenting 1-related protein kinases 2 activated by hyperosmotic and saline stresses in *Arabidopsis thaliana*. J Biol Chem.

[23] Khan MR (2020). Genome-wide identification and expression analysis of SnRK2 gene family in mungbean (*Vigna radiata*) in response to drought stress. Crop Pasture Sci.

[24] Liu Z, Ge X, Yang Z, Zhang C, Zhao G, Chen E, Liu J, Zhang X, Fuguang L (2017). Genome-wide identification and characterization of SnRK2 gene family in cotton (Gossypium hirsutum L.). BMC Genetics..

[25] Shao Y, Qin Y, Zou Y, Ma F (2014). Genome-wide identification and expression profiling of the *SnRK2* gene family in *Malus prunifolia*. Gene.

[26] Shen X, Guo X, Zhao D, Zhang Q, Jiang Y, Wang Y, Peng X, Wei Y, Zhai Z, Zhao W, Li T (2017). Cloning and expression profiling of the *PacSnRK2* and *PacPP2C* gene families during fruit development, ABA treatment, and dehydration stress in sweet cherry. Plant Physiol Biochem.

[27] Song X, Ohtani M, Hori C, Takebayasi A, Qiang Z (2015). Physical interaction between *SnRK2* and *PP2C* is conserved in *Populus trichocarpa*. Plant Biotechnology.

[28] Yamaguchi-Shinozaki K (2010). Molecular Basis of the Core Regulatory Network in ABA Responses: Sensing, Signaling and Transport. Plant Cell Physiol.

[29] Boudsocq M, Droillard MJ, Barbier-Brygoo H, Laurière C (2007). Different phosphorylation mechanisms are involved in the activation of sucrose non-fermenting 1 related protein kinases 2 by osmotic stresses and abscisic acid. Plant Mol Biol.

[30] Zhang F, Chen XJ, Wang JH, Zheng J (2015). Overexpression of a maize SNF-related protein kinase gene, ZmSnRK2.11, reduces salt and drought tolerance in Arabidopsis. J Integr Agric.

[31] Diédhiou CJ, Popova OV, Dietz KJ, Golldack D (2008). The SNF1-type serine-threonine protein kinase SAPK4 regulates stress-responsive gene expression in rice. BMC Plant Biol.

[32] Tian S, Mao X, Zhang H, Chen S, Zhai C, Yang S, Jing R (2013). Cloning and characterization of TaSnRK2.3, a novel SnRK2 gene in common wheat. J Exp Bot.

[33] Zou C, Chen A, Xiao L, Muller HM, Ache P, Haberer G, Zhang M, Jia W, Deng P, Huang R, Lang D, Li F, Zhan D, Wu X, Zhang H, Bohm J, Liu R, Shabala S, Hedrich R, Zhu JK, Zhang H (2017). A high-quality genome assembly of quinoa provides insights into the molecular basis of salt bladder-based salinity tolerance and the exceptional nutritional value. Cell Res.

[34] Jarvis DE, Ho YS, Lightfoot DJ, Schmöckel SM, Li B, Borm TJ, Ohyanagi H, Mineta K, Michell CT, Saber N, Kharbatia NM, Rupper RR, Sharp AR, Dally N, Boughton BA, Woo YH, Gao G, Schijlen EG, Guo X, Momin AA, Negrão S, Al-Babili S, Gehring C, Roessner U, Jung C, Murphy K, Arold ST, Gojobori T, Linden CG, van Loo EN, Jellen EN, Maughan PJ, Tester M (2017). The genome of *Chenopodium quinoa*. Nature.

[35] Morales A, Zurita-Silva A, Maldonado J, Silva H (2017). Transcriptional Responses of Chilean Quinoa (Chenopodium quinoa Willd.) Under Water Deficit Conditions Uncovers ABA-Independent Expression Patterns. Front Plant Sci.

[36] Hongying Z, Ruilian J, Xinguo M (2017). Functional Characterization of TaSnRK2.8 Promoter in Response to Abiotic Stresses by Deletion Analysis in Transgenic Arabidopsis. Front Plant Sci.

[37] Lee HJ, Park YJ, Seo PJ, Kim JH, Sim HJ, Kim SG, Park CM (2015). Systemic Immunity Requires SnRK2.8-Mediated Nuclear Import of NPR1 in Arabidopsis. Plant Cell.

[38] Yoshida R, Umezawa T, Mizoguchi T, Takahashi S, Takahashi F, Shinozaki K (2006). The Regulatory Domain of SRK2E/OST1/SnRK2.6 Interacts with ABI1 and Integrates Abscisic Acid (ABA) and Osmotic Stress Signals Controlling Stomatal Closure in Arabidopsis. J Biol Chem.

[39] Fujita Y, Yoshida T, Yamaguchi-Shinozaki K (2013). Pivotal role of the AREB/ABF-SnRK2 pathway in ABRE-mediated transcription in response to osmotic stress in plants. Physiol Plant.

[40] Ghillebert R, Swinnen E, Jing W, Vandesteene L, Ramon M, Norga K, Rolland F, Winderickx J (2011). The AMPK/SNF1/SnRK1 fuel gauge and energy regulator: structure, function and regulation. FEBS J.

[41] Zhang H, Li W, Mao X, Jing R, Jia H (2016). Differential Activation of the Wheat *SnRK2* Family by Abiotic Stresses. Front Plant Sci.

[42] Abdullah, Faraji S, Mehmood F, Malik H, Poczai P. The GASA Gene Family in Theobroma cacao: Genome Wide Identification and Expression Analyses. Agronomy. 2021;11(1):1–38.

[43] Musavizadeh Z, Najafi-Zarrini H, Kazemitabar SK, Hashemi SH, Faraji S, Barcaccia G, Heidari P (2021). Genome-Wide Analysis of Potassium Channel Genes in Rice: Expression of the OsAKT and OsKAT Genes under Salt Stress. Genes.

[44] Heidari P, Faraji S, Ahmadizadeh M, Ahmar S, Mora-Poblete F (2021). New Insights Into Structure and Function of *TIFY* Genes in *Zea mays* and *Solanum lycopersicum*: A Genome-Wide Comprehensive Analysis. Front Genet.

[45] Faraji S, Heidari P, Amouei H, Filiz E, Abdullah, Poczai P. Investigation and Computational Analysis of the Sulfotransferase (SOT) Gene Family in Potato (Solanum tuberosum): Insights into Sulfur Adjustment for Proper Development and Stimuli Responses. Plants (Basel). 2021;10(12):2597–619. 10.3390/plants10122597 PMC8707064 34961068

[46] Wang MM, Liu MM, Ran F, Guo PC, Ke YZ, Wu YW, Wen J, Li PF, Li JN, Du H (2018). Global Analysis of WOX Transcription Factor Gene Family in *Brassica napus* Reveals Their Stress- and Hormone-Responsive Patterns. Int J Mol Sci.

[47] Fujii H, Zhu JK (2009). Arabidopsis mutant deficient in 3 abscisic acid-activated protein kinases reveals critical roles in growth, reproduction, and stress. Proc Natl Acad Sci.

[48] Yue M, Szostkiewicz I, Korte A, Moes D, Yi Y, Christmann A, Grill E (2009). Regulators of PP2C Phosphatase Activity Function as Abscisic Acid Sensors. Science.

[49] Nakashima K, Fujita Y, Kanamori N, Katagiri T, Umezawa T, Kidokoro S, Maruyama K, Yoshida T, Ishiyama K, Kobayashi M, Shinozaki K, Yamaguchi-Shinozaki K (2009). Three Arabidopsis SnRK2 Protein Kinases, SRK2D/SnRK2.2, SRK2E/SnRK2.6/OST1 and SRK2I/SnRK2.3, Involved in ABA Signaling are Essential for the Control of Seed Development and Dormancy. Plant Cell Physiol.

[50] Mao X, Zhang H, Tian S, Chang X, Jing R (2010). TaSnRK2.4, an SNF1-type serine/threonine protein kinase of wheat (Triticum aestivum L.), confers enhanced multistress tolerance in Arabidopsis. J Exp Bot.

[51] Shin R, Alvarez S, Burch AY, Jez JM, Schachtman DP (2007). Phosphoproteomic identification of targets of the Arabidopsis sucrose nonfermenting-like kinase SnRK2.8 reveals a connection to metabolic processes. PNAS..

[52] Li J (1996). An Abscisic Acid-Activated and Calcium-Independent Protein Kinase from Guard Cells of Fava Bean. Plant Cell.

[53] Baradaran A, Sieo CC, Foo HL, Illias RM, Yusoff K, Rahim RA (2013). Cloning and in silico characterization of two signal peptides from *Pediococcus pentosaceus* and their function for the secretion of heterologous protein in *Lactococcus lactis*. Biotech Lett.

[54] Gardy JL, Laird MR, Chen F, Rey S, Walsh CJ, Ester M (2005). PSORTb v.2.0: expanded prediction of bacterial protein subcellular localization and insights gained from comparative proteome analysis. Bioinformatics..

[55] Larkin MA, Blackshields G, Brown NP, Chenna R, McGettigan PA, McWilliam H (2007). Clustal W and Clustal X version 2.0. Bioinformatics.

[56] Chen CJ, Chen H, Zhang Y, Thomas HR, Frank MH, He YH (2020). TBtools, a toolkit for biologists integrating various HTS-datahandling tools with a user-friendly interface. Mol Plant.

[57] Steven DC (1996). Molecular genetic studies confirm the role of brassinosteroids in plant growth and development. Plant J.

[58] Szklarczyk D, Franceschini A, Wyder S, Forslund K, Heller D, Huerta-Cepas J (2015). STRING v10: Protein-protein interaction networks, integrated over the tree of life. Nucl Acids Res..

[59] Lescot M, Dehais P, Thijs G, Marchal K, Moreau Y, Van PY (2002). PlantCARE, a database of plant cis-acting regulatory elements and a portal to tools for in silico analysis of promoter sequences. Nucl Acids Res.

[60] Gao X, Chen Y, Chen M, Wang S, Wen X, Zhang S (2018). Identification of key candidate genes and biological pathways in bladder cancer. PeerJ.

[61] Livak KJ, Schmittgen TD (2002). Analysis of Relative Gene Expression Data using Real-Time Quantitative PCR. Methods.

