# Supplementary figures and images for: Identification and expression analysis of the CqSnRK2 gene family and a functional study of the CqSnRK2.12 gene in quinoa (Chenopodium quinoa Willd.)
Source: BMC Genomics. 2022 May 24;23:397. doi: 10.1186/s12864-022-08626-1 (PMC9131629; doi:10.1186/s12864-022-08626-1)

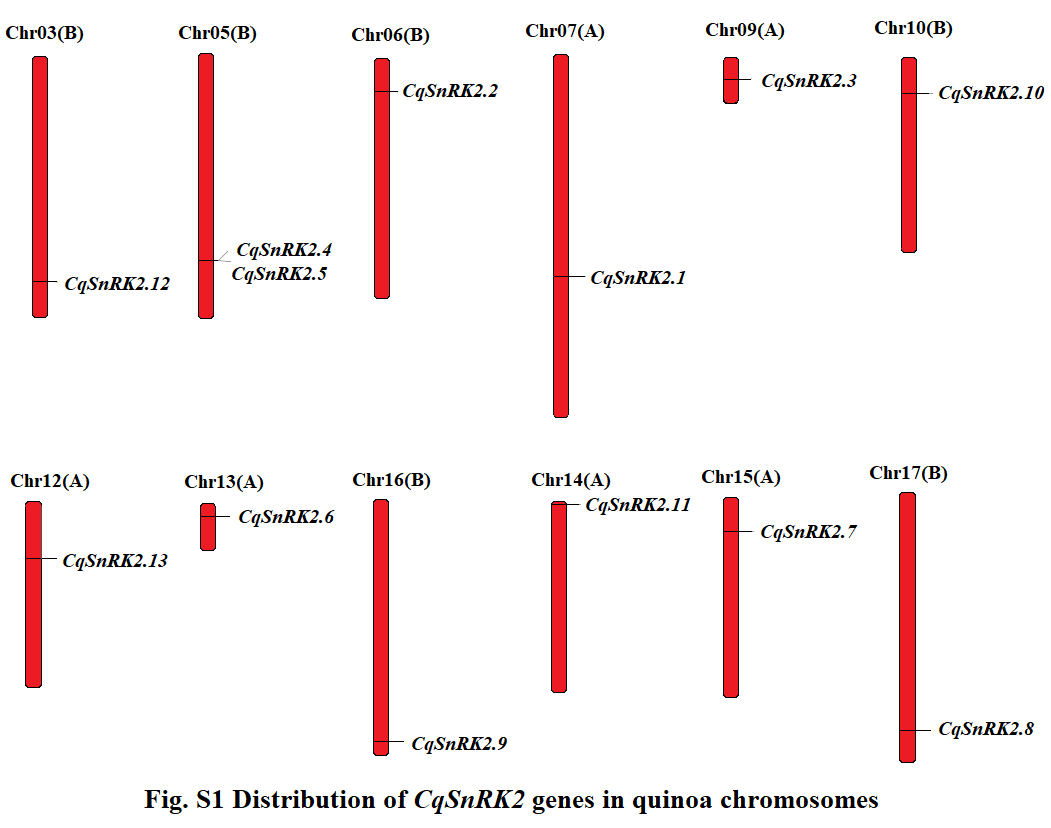

Supplement: Supplementary file 1 — Additional file 1. [file 12864_2022_8626_MOESM1_ESM.tif]

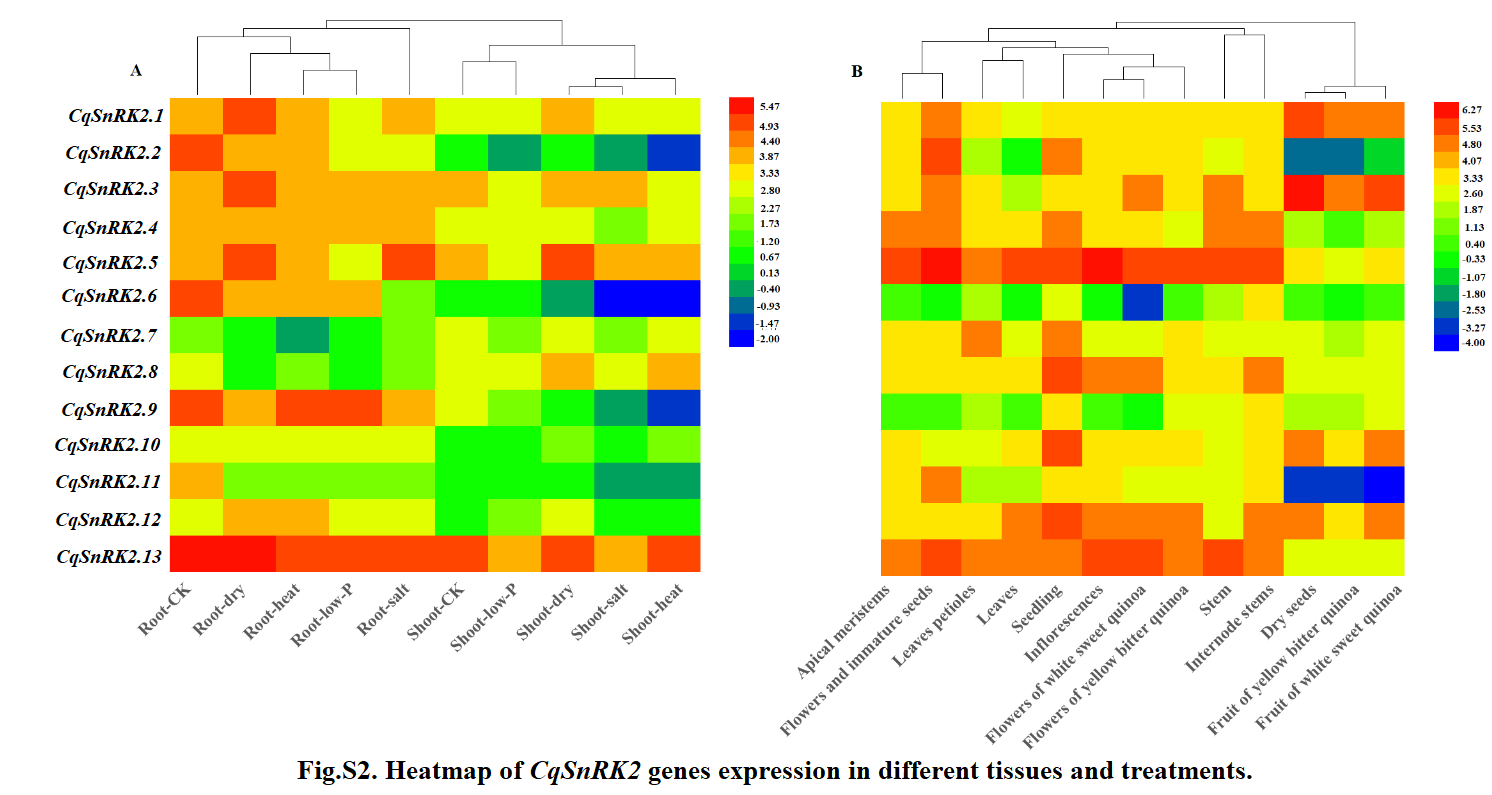

Supplement: Supplementary file 2 — Additional file 2. [file 12864_2022_8626_MOESM2_ESM.tif]
